# Systematics and biology of some species of *Micrurapteryx* Spuler (Lepidoptera, Gracillariidae) from the Holarctic Region, with re-description of *M. caraganella* (Hering) from Siberia

**DOI:** 10.3897/zookeys.579.7166

**Published:** 2016-04-11

**Authors:** Natalia Kirichenko, Paolo Triberti, Marko Mutanen, Emmanuelle Magnoux, Jean-François Landry, Carlos Lopez-Vaamonde

**Affiliations:** 1 Sukachev Institute of Forest SB RAS, Akademgorodok 50/28, 660036, Krasnoyarsk, Russia; 2 Siberian Federal University, 79 Svobodny pr., 660041, Krasnoyarsk, Russia; 3 INRA, UR0633 Zoologie Forestière, F-45075 Orléans, France; 4 Museo Civico di Storia Naturale, Lungadige Porta Vittoria 9, I37129, Verona, Italy; 5 Department of Genetics and Physiology, P.O. Box 3000, FI-90014 University of Oulu, Finland; 6 Agriculture and Agri-Food Canada, Ottawa Research and Development Centre, Central Experimental Farm, Ottawa, Ontario K1A 0C6, Canada; 7 Institut de Recherche sur la Biologie de l’Insecte, CNRS UMR 7261, Université François-Rabelais de Tours, UFR Sciences et Techniques, 37200 Tours, France

**Keywords:** Leaf-mining moth, Micrurapteryx caraganella, Micrurapteryx gradatella, Micrurapteryx occulta, Parectopa albicostella, Siberian peashrub, COI, histone H3, 28S, Canada, USA

## Abstract

During a DNA barcoding campaign of leaf-mining insects from Siberia, a genetically divergent lineage of a gracillariid belonging to the genus *Micrurapteryx* was discovered, whose larvae developed on *Caragana* Fabr. and *Medicago* L. (Fabaceae). Specimens from Siberia showed similar external morphology to the Palearctic *Micrurapteryx gradatella* and the Nearctic *Parectopa occulta* but differed in male genitalia, DNA barcodes, and nuclear genes histone H3 and 28S. Members of this lineage are re-described here as *Micrurapteryx caraganella* (Hering, 1957), **comb. n.**, an available name published with only a brief description of its larva and leaf mine.

*Micrurapteryx caraganella* is widely distributed throughout Siberia, from Tyumen oblast in the West to Transbaikalia in the East. Occasionally it may severely affect its main host, *Caragana arborescens* Lam. This species has been confused in the past with *Micrurapteryx gradatella* in Siberia, but field observations confirm that *Micrurapteryx gradatella* exists in Siberia and is sympatric with *Micrurapteryx caraganella*, at least in the Krasnoyarsk region, where it feeds on different host plants (*Vicia amoena* Fisch. and *Vicia* sp.).

In addition, based on both morphological and molecular evidence as well as examination of type specimens, the North American *Parectopa occulta* Braun, 1922 and *Parectopa albicostella* Braun, 1925 are transferred to *Micrurapteryx* as *Micrurapteryx occulta* (Braun, 1922), **comb. n.** with *albicostella* as its junior synonym (**syn. n.**). Characters used to distinguish *Micrurapteryx* from *Parectopa* are presented and illustrated. These findings provide another example of the potential of DNA barcoding to reveal overlooked species and illuminate nomenclatural problems.

## Introduction

With more than 2000 described species, the family Gracillariidae represents one of the most diverse groups of small moths (De Prins and De Prins 2015). Many species of gracillariids remain to be discovered, especially in the tropical regions (Lees et al. 2013; Brito et al. 2013) but also in the Palearctic (Laštůvka et al. 2013; Kobayashi et al. 2013; Kirichenko et al. 2015) and Nearctic regions (Davis and Deschka 2001).

The genus *Micrurapteryx* Spuler, 1910, contains 11 species all distributed in the Holarctic Region (De Prins and De Prins 2015). Ten species occur in the Palearctic Region: *Micrurapteryx bidentata* Noreika, 1992, *Micrurapteryx fumosella* Kuznetzov & Tristan, 1985, *Micrurapteryx gerasimovi* Ermolaev, 1982, *Micrurapteryx gradatella* (Herrich-Schäffer, 1855), *Micrurapteryx kollariella* (Zeller, 1839), *Micrurapteryx parvula* Amsel, 1935, *Micrurapteryx sophorella* Kuznetzov, 1979, *Micrurapteryx sophorivora* Kuznetzov & Tristan, 1985, *Micrurapteryx tibetiensis* Bai & Li, 2013, and *Micrurapteryx tortuosella* Kuznetzov & Tristan, 1985. Larvae of six species mine the leaves of legumes (Fabaceae). Five species feed on up to four different legume genera (*Astragalus* L., *Lathyrus* L., *Medicago* L., *Melilotus* L., *Sophora* L., *Robinia* L., *Trifolium* L. and *Vicia* L.) (Dovnar-Zapol’skiy 1969; Kuznetzov and Tristan 1985; Barakanova 1986; Ermolaev 1982; Gencer and Seven 2005; De Prins and De Prins 2015) (see Suppl. material 1: Table S1). As an exception, *Micrurapteryx kollariella* has been recorded mining leaves of eleven legume genera (Suppl. material 1: Table S1). For four species *Micrurapteryx bidentata*, *Micrurapteryx parvula*, *Micrurapteryx sophorella* and *Micrurapteryx tibetiensis* hosts remain unknown (Kuznetzov and Tristan 1985; Noreika and Puplesis 1992; Bai 2013). Only one species has been recorded from the Nearctic Region, *Micrurapteryx salicifoliella* (Chambers, 1872), whose larvae mine leaves of *Salix* (De Prins and De Prins 2015).

During a DNA barcoding campaign of leaf-mining insects from Siberia carried out in 2011, we discovered a genetically divergent lineage of *Micrurapteryx* feeding on the Siberian peashrub *Caragana arborescens* (Fabaceae). Preliminary barcoding data showed pronounced divergence in the COI barcoding fragment from European specimens of *Micrurapteryx gradatella*. Examination of the genitalia revealed that it was clearly different from European *Micrurapteryx gradatella*.

In their taxonomic review of the Palearctic *Micrurapteryx*, Kuznetzov and Tristan (1985) called *Micrurapteryx gradatella* the species found in Siberia mining “yellow acacia” (= *Caragana arborescens*). They also stated that despite the confusion in the Russian literature about various names applied to specimens mining *Caragana* in Siberia, in their estimation there was only one species present, which they deemed to be *Micrurapteryx gradatella*. Subsequent works (Noreika 1997; Kuznetzov and Baryshnikova 1998; Kuznetzov 1999) followed Kuznetzov and Tristan (1985).

Contrary to these authors, our findings indicated unequivocally that at least two species were present. This raised the question of whether the *Caragana*-feeding lineage from Siberia represented an undescribed *Micrurapteryx* species. Two unavailable names have been used in the literature to refer to a species feeding on *Caragana arborescens* in Siberia: *Parectopa caraganella* Danilevsky and *Parectopa caraginella* Danilevsky (Hering 1957; Dovnar-Zapol’skiy 1969; Kuznetzov and Tristan 1985; De Prins and De Prins 2015). The lingering confusion about the identity of *Caragana*-feeding *Micrurapteryx* in Siberia is partly due to the lack of a detailed description of *Micrurapteryx gradatella* in Europe and an over-reliance on wing pattern characters without examination of genitalia. Only recently both female and male genitalia of *Micrurapteryx gradatella* have been illustrated (Bengtsson and Johansson 2011), but that description was very brief.

Based on differences in morphology and DNA sequence data (mitochondrial and nuclear), we assess that there are two species of *Micrurapteryx* in Siberia, *Micrurapteryx caraganella* and *Micrurapteryx gradatella*. We present elaborated morphological re-descriptions of the adults of both species. In addition, we compare the morphology and DNA barcodes with other European and North American *Micrurapteryx*, as well as some related species of *Parectopa* developing on Fabaceae whose barcodes clustered near *Micrurapteryx*.

The availability of the binomen *Micrurapteryx caraganella* with authorship attributed to Hering (1957) is discussed. We show that the Nearctic *Parectopa occulta* Braun, 1922 in fact belongs to *Micrurapteryx* (comb. n.) and is closely related to the Palearctic *Micrurapteryx gradatella*, and is re-described. In addition, based on examination of type specimens the North American *Parectopa albicostella* Braun, 1925 is shown to be a junior synonym (syn. n.) of *Micrurapteryx occulta*. Finally an assessment of morphological characters are presented that distinguish *Micrurapteryx* from *Parectopa*.

## Material and methods

### Sampling

Leaf mines of *Micrurapteryx caraganella* were collected on *Caragana arborescens* at eight administrative regions in Siberia: in Novosibirsk oblast (Novosibirsk: Central Siberian botanical garden SB RAS, June-July 2011–2013, July 2015), Krasnoyarsk krai (Krasnoyarsk: Akademgorodok, the left bank of the river Yenisei, June-August 2013–2014, July 2015), Omsk oblast (Omsk: Victory park, city plantations, June 2013, July 2015), Tyumen oblast (Tyumen: Zatyumenskiy park; Tobolsk: Ermak garden, July 2015), Altai krai (Barnaul: Izymrudniy park, July 2015), Irkutsk oblast (Irkutsk: dendropark of the ethnographic museum “Talcy”, August 2015), Republic of Buryatia (Ulan-Ude: Smolina street, August 2015) and Transbaikal krai (Chita: Victory Park, August 2015). Thus, in all cases sampling was done in urban ecosystems, on planted bushes of *Caragana* spp., on *Caragana arborescens* in all localities, additionally on *Caragana frutex* (L.) K. Koch and *Medicago sativa* L. in Omsk and *Caragana boisii* C. K. Schneid. in Novosibirsk. In all localities, except two, both the damaged leaves (carrying mines) and live insects (larvae in mines or pupae in cocoons on leaves) were collected; in Ulan-Ude and Chita, only empty mines were found which were preserved as herbarium vouchers. For comparative purposes, in early July 2015 we also collected mines with live larvae of *Micrurapteryx gradatella* on *Vicia amoena* in suburb of Krasnoyarsk (Yenisei river bank, near Karaulnaya biostation) and *Parectopa ononidis* (Zeller, 1839) on *Trifolium pratense* L. in suburb of Krasnoyarsk (Yenisei river bank, Skala Berkut).

Mined leaflets as well as larvae feeding in mines and pupating on leaves were photographed in nature and in the laboratory with a digital camera Sony Nex3 (in laboratory, the photographs were taken through a Zeiss STEMI DV4 binocular microscope).

Adults of *Micrurapteryx caraganella* examined in this study were obtained by rearing larvae and pupae collected on *Caragana arborescens* in July-August 2013–2015 and on *Caragana frutex* in July 2015. Six larvae and seven pupae were preserved in 96% ethanol, including a specimen on *Caragana boisii*, for genetic and morphological analyses. In addition, 70 larvae were left to complete their development in glass jars (200 ml) lined with filter paper on the bottom, in laboratory conditions (22 °C, 55% RH, LD 18:6 h photoperiod). As leaflets of the host plant dry quickly, mined leaflets were collected with a short section of twig; the latter was tightly wrapped in paper tissue and moisturized every second day, following guidelines of Ohshima (2005). Twelve pupated larvae, collected in nature as well as those that pupated in the laboratory, were transferred to Petri dishes (90 mm in diameter) on filter paper and kept until the adults emerged. In the Petri dishes, the humidity was regulated by adding few drops of water to a small cotton ball attached inside the lid. In total 32 larvae out of 70 larvae pupated and 30 adults emerged. Larvae of *Micrurapteryx gradatella* (5 specimens) and *Parectopa ononidis* (4), collected near Krasnoyarsk, were grown in the same conditions as above and 2 adults of each species emerged.

Samples of *Micrurapteryx salicifoliella*, *Micrurapteryx occulta*, *Parectopa lespedezaefoliella* Clemens, 1860 and *Parectopa robiniella* Clemens, 1863 from North America, as well as *Micrurapteryx gradatella* and *Micrurapteryx kollariella* from Europe were also examined. All specimens used in this study for both genetic and morphological analyses are listed in Tables 1 and Suppl. material 1: Table S2.

**Table 1.** Specimens used for molecular analyses. Both the Process ID and Sample ID codes are unique identifiers linking the record in the BOLD database and the voucher specimen from which the sequence is derived. Additional collecting and specimen data are accessible in the BOLD dataset dx.doi.org/10.5883/DS-MICRURA as well as GenBank (http://www.ncbi.nlm.nih.gov/genbank/). Where pertinent, genitalia preparation number and sex are given in square brackets in the Sample ID column.

№	Sample ID and genitalia preparation in []	Process ID	Host plant	Country	GenBank accession COI	GenBank accession H3	GenBank accession 28S
***Micrurapteryx caraganella***							
1	NK58	GRPAL1102-13	*Caragana boisii*	Russia	KP845396	KP856945	KP845432
2	NK189, [TRB3986♀]	ISSIK234-14	*Caragana arborescens*	Russia	KP845393	KP856944	KP845431
3	NK414	ISSIK363-14	*Caragana arborescens*	Russia	KP845397	KP856946	KP845433
4	NK415, [TRB4061♀]	ISSIK364-14	*Caragana arborescens*	Russia	KP845405	KP856950	KP845437
5	NK416	ISSIK365-14	*Caragana arborescens*	Russia	KP845402	KP856948	KP845435
6	NK417	ISSIK366-14	*Caragana arborescens*	Russia	KP845424	KP856959	KP845445
7	NK418	ISSIK367-14	*Caragana arborescens*	Russia	KP845391	KP856943	KP845430
8	NK429	MICRU001-15	*Caragana arborescens*	Russia	KP845418	KP856957	KP845443
9	NK430	MICRU002-15	*Caragana arborescens*	Russia	KP845400	KP856947	KP845434
10	NK431	MICRU003-15	*Caragana arborescens*	Russia	KP845415	KP856955	KP845442
11	NK432	MICRU004-15	*Caragana arborescens*	Russia	KP845389	KP856942	KP845429
12	NK433, [TRB3994♂]	MICRU005-15	*Caragana arborescens*	Russia	KP845387	KP856941	KP845428
13	NK434, [TRB4052♀]	MICRU006-15	*Caragana arborescens*	Russia	KP845425	KP856960	KP845446
14	NK439	MICRU011-15	*Caragana arborescens*	Russia	–	KP856951	KP845438
15	NK470	MICRU025-15	*Medicago* sp.	Russia	KU380252	KU380277	KU380273
16	NK472	MICRU027-15	*Caragana arborescens*	Russia	KU380260	KU380278	KU380274
17	NK473	MICRU028-15	*Caragana arborescens*	Russia	KU380247	KU380275	KU380271
18	NK474	MICRU029-15	*Caragana arborescens*	Russia	KU380268	–	–
19	NK475	MICRU030-15	*Caragana arborescens*	Russia	KU380254	–	–
20	NK476	MICRU031-15	*Caragana arborescens*	Russia	KU380246	–	–
21	NK477	MICRU032-15	*Caragana arborescens*	Russia	KU380257	–	–
22	NK478	MICRU033-15	*Caragana arborescens*	Russia	KU380267	–	–
***Micrurapteryx gradatella***							
23	MM08526	LEFIE211-10	*Lathyrus linifolius*	Finland	HM873950	–	–
24	MM15541	LEFIG677-10	–	Finland	HM876337	–	–
25	MM18085	LEFIK510-10	–	Finland	JF854112	–	–
26	NK435	MICRU007-15	*Lathyrus linifolius*	Finland	KP845413	KP856953	KP845440
27	NK436	MICRU008-15	*Lathyrus linifolius*	Finland	KP845411	KP856952	KP845439
28	NK437	MICRU009-15	*Lathyrus linifolius*	Finland	KP845403	KP856949	KP845436
29	NK438	MICRU010-15	*Lathyrus linifolius*	Finland	KP845414	KP856954	KP845441
30	NK440	MICRU012-15	*Lathyrus linifolius*	Finland	–	KP856958	KP845444
31	NK459	MICRU014-15	*Vicia amoena*	Russia	KU380248	KU380276	KU380272
32	NK462	MICRU017-5	*Vicia amoena*	Russia	KU380266		
33	NK471	MICRU026-15	*Vicia amoena*	Russia	KU380245		
***Micrurapteryx kollariella***							
34	CLV1781	GRSLO261-10	–	Austria	JF848362	–	–
35	CLV1832	GRSLO312-10	–	Italy	JF848397	–	–
36	CLV2281	GRPAL123-11	–	France	KP845406	–	–
37	CLV5200	LNOUD2104-12	–	Romania	KP845417	–	–
38	TLMF Lep 03523	PHLAD348-11	–	France	KP845404	–	–
39	TLMF Lep 03534	PHLAD359-11	–	Italy	JN272048	–	–
***Micrurapteryx occulta***							
40	CNCLEP00008459, [MIC6944♂]	MNAL461-10	–	USA	HQ965133	–	–
41	CNCLEP00035771, [MIC6945♂]	MNAL496-10	–	Canada	HQ965158	–	–
42	CNCLEP00035785, [MIC6938♂]	MNAL498-10	–	Canada	HQ965160	–	–
43	CNCLEP00038523, [MIC6839♂]	MNAI744-09	–	Canada	GU692590	–	–
44	CNCLEP00082614, [MIC6943♂]	MNAN395-11	–	USA	JN272038	–	–
45	CNCLEP00082615, [MIC6953♂]	MNAN396-11	–	USA	JN272039	–	–
46	CNCLEP00082616, [MIC6954♂]	MNAN397-11	–	USA	JN272040	–	–
47	CNCLEP00082676, [MIC6937♂]	MNAN400-11	–	USA	JN272042	–	–
48	EDL YAKIMALUPINEA 1Jun2011	EHL942-12	–	USA	KP845419	–	–
49	USNMENT00657162, [USNM130246♂]	MNAM941-10	*Lathyrus* sp.	USA	JN272015	–	–
50	USNMENT00657163, [USNM130247♂]	MNAM942-10	*Lathyrus* sp.	USA	JN272016	–	–
51	USNMENT00657165, [USNM130248♂]	MNAM944-10	*Lathyrus* sp.	USA	JN272017	–	–
52	jflandry1800 =CNCLEP00016559, [MIC6901♀]	MECB818-05	–	Canada	KP845423	–	–
53	jflandry1801 =CNCLEP00016560, [MIC6955♀]	MECB819-05	–	Canada	KP845422	–	–
54	jflandry1804 =CNCLEP00016563, [MIC6956♀]	MECB822-05	–	Canada	KP845408	–	–
55	CNCLEP00121158, [MIC 6904♀]	MNAQ068-15	*Lupinus* sp.	Canada	KU380256	–	–
56	CNCLEP00121159, [MIC6905♂]	MNAQ069-15	*Lupinus* sp.	Canada	KU380261	–	–
57	AC006119, [MIC6948♂]	MNAQ382-15	–	Canada	KU380255	–	–
58	AC006629, [MIC 6946♂]	MNAQ385-15	–	Canada	KU380244	–	–
59	CNCLEP00108894, [MIC6949 ♂]	MNAQ402-15	–	Canada	KU380265	–	–
60	CNCLEP00076976, [MIC 6947 ♂]	MNAQ392-15	–	USA	KU380263	–	–
61	AC006130, [MIC6939♂]	MNAQ384-15	–	Canada	KU380262	–	–
62	BIOUG16843-E11	CNIVB1119-14	–	Canada	KT131992	–	–
63	BIOUG16843-E08, [MIC7558♂]	CNIVB1116-14	–	Canada	KT147247	–	–
64	BIOUG16843-E05, [MIC7459♀]	CNIVB1113-14	–	Canada	KT133090	–	–
65	BIOUG16843-E04 [MIC7562♀]	CNIVB1112-14	–	Canada	KT142702	–	–
66	BIOUG16843-E02, [MIC7456♂]	CNIVB1110-14	–	Canada	KT141504	–	–
67	BIOUG16790-A06	CNIVA638-14	–	Canada	KT145371	–	–
68	BIOUG16148-A09	SMTPJ2503-14	–	Canada	KT138035	–	–
69	BIOUG16138-A01, [MIC7457♂]	SMTPJ1378-14	–	Canada	KT126913	–	–
70	BIOUG16087-B07	SMTPI8811-14	–	Canada	KT131533	–	–
71	BIOUG16013-G08	SMTPI2530-14	–	Canada	KT147946	–	–
72	BIOUG10643-A09	CNGBJ1629-14	–	Canada	KR454708	–	–
73	BIOUG09474-A06, [MIC7554♂)]	CNGMA1885-13	–	Canada	KR451687	–	–
74	BIOUG09363-F01	CNGBB550-13	–	Canada	KR450358	–	–
75	BIOUG08486-H06, [MIC7561♂]	SSWLE3847-13	–	Canada	KM541048	–	–
76	BIOUG08285-E05, [MIC7460♀]	SSPAC6698-13	–	Canada	KM542253	–	–
77	BIOUG08285-A11, [MIC7555♀]	SSPAC6656-13	–	Canada	KM553942	–	–
78	BIOUG07668-H10	NGNAG247-13	–	Canada	KT137773	–	–
79	BIOUG07512-G07	NGNAD1517-13	–	Canada	KT139585	–	–
80	BIOUG07391-H10	NGNAC3018-13	–	Canada	KT128577	–	–
81	BIOUG07213-F11	NGNAB1279-13	–	Canada	KT134205	–	–
82	BIOUG07213-E07	NGNAB1263-13	–	Canada	KT142705	–	–
83	BIOUG07133-F02	NGNAA1737-13	–	Canada	KT142617	–	–
84	BIOUG21939-G09	SMTPL3504-15	–	Canada	KU380264	–	–
85	BIOUG07133-D05	NGNAA1716-13	–	Canada	KT139942	–	–
86	BIOUG07047-G04	NGNAA361-13	–	Canada	KT144572	–	–
87	BIOUG06814-D03, [MIC7559♀]	CNWLM079-13	–	Canada	KM544224	–	–
88	BIOUG06714-A06, [MIC7455♂]	JMMMB449-13	–	United States	KU380251	–	–
89	BIOUG05675-G12	SMTPB16614-13	–	Canada	KT141098	–	–
90	BIOUG05658-H08	SMTPB15007-13	–	Canada	KR936951	–	–
91	BIOUG05658-H07	SMTPB15006-13	–	Canada	KT140585	–	–
92	BIOUG05658-H06	SMTPB15005-13	–	Canada	KT136403	–	–
93	BIOUG05528-B12	SMTPB2589-13	–	Canada	KT143475	–	–
94	BIOUG03957-A01, [MIC7557♀]	CNRMF4146-12	–	Canada	KM547661	–	–
95	BIOUG03754-B12, [MIC7556♀]	CNRMF2498-12	–	Canada	KM547518	–	–
96	BIOUG03484-B11, MIC7458♂]	CNWLF184-12	–	Canada	KM542391	–	–
97	BIOUG03017-H02, [MIC7553♂]	CNRMA371-12	–	Canada	KM548929	–	–
98	BIOUG02884-D02, [MIC7560♂]	CNJAA025-12	–	Canada	KM540469	–	–
99	BIOUG07133-D08	NGNAA1719-13	–	Canada	KT125110	–	–
100	BIOUG21903-F08	SMTPL296-15	–	Canada	KU380250	–	–
101	BIOUG20492-G06	CNTIA1902-15	–	Canada	KU380249	–	–
102	BIOUG20492-F11	CNTIA1895-15	–	Canada	KU380253	–	–
103	BIOUG18949-E06	CNYOA518-15	–	Canada	KR936641	–	–
104	BIOUG18164-F07	CNKTC1685-15	–	Canada	KT131089	–	–
105	BIOUG17972-E10	CNKTB2181-14	–	Canada	KT147497	–	–
106	BIOUG17786-F09	CNKTA1035-14	–	Canada	KT147730	–	–
107	BIOUG17786-F07	CNKTA1033-14	–	Canada	KT132114	–	–
108	BIOUG17786-F06	CNKTA1032-14	–	Canada	KT141434	–	–
109	BIOUG17786-F05	CNKTA1031-14	–	Canada	KT132493	–	–
110	BIOUG17245-D09	CNKLA840-14	–	Canada	KT143953	–	–
111	BIOUG16989-D12	CNIVF402-14	–	Canada	KT131234	–	–
112	BIOUG16944-A01	CNIVE102-14	–	Canada	KT126687	–	–
***Micrurapteryx salicifoliella***							
113	10BBCLP-2121	BBLPD123-10	–	Canada	KM546499	–	–
114	10BBCLP-2122	BBLPD124-10	–	Canada	KM551613	–	–
115	10BBCLP-2123	BBLPD125-10	–	Canada	KM544406	–	–
116	10BBCLP-2125	BBLPD127-10	–	Canada	KM542568	–	–
117	10BBCLP-2126	BBLPD128-10	–	Canada	KM539529	–	–
118	10BBCLP-2129	BBLPD131-10	–	Canada	KM550976	–	–
119	10BBCLP-2130	BBLPD132-10	–	Canada	KM553079	–	–
120	10BBCLP-2131 [MIC7454♂]	BBLPD133-10	–	Canada	KM542107	–	–
121	10BBCLP-2132	BBLPD134-10	–	Canada	KM549534	–	–
122	10BBCLP-2133	BBLPD135-10	–	Canada	KM547436	–	–
123	10PROBE-18724	EMHLC005-10	–	Canada	HQ946212	–	–
124	10PROBE-18785	EMHLC046-10	*Salix* sp.	Canada	HQ946239	–	–
125	10PROBE-19679	EMHLC162-10	*Salix* sp.	Canada	HQ946317	–	–
126	10PROBE-19681	EMHLC164-10	*Salix* sp.	Canada	HQ946318	–	–
127	10PROBE-21923	PHLCH266-10	–	Canada	JF860432	–	–
128	10PROBE-25766	PHLCH349-10	*Myrica gale*	Canada	JF860441	–	–
129	AC005056, [MIC6840♂]	LQAC045-06	–	Canada	KP845395	–	–
130	BIOUG03504-A05	SSBAA5768-12	–	Canada	KM548123	–	–
131	BIOUG04663-C02	SSJAB037-13	–	Canada	KM550643	–	–
132	BIOUG04663-C03	SSJAB038-13	–	Canada	KM551664	–	–
133	BIOUG04663-D07	SSJAB054-13	–	Canada	KM541113	–	–
134	BIOUG04722-F07	SSJAA015-13	–	Canada	KM550409	–	–
135	BIOUG05528-B11	SMTPB2588-13	–	Canada	KP845407	–	–
136	BIOUG06046-B12	SSJAC213-13	–	Canada	KM543829	–	–
137	HLC-10432	XAF391-05	–	Canada	KP845420	–	–
138	KENWR 7198	ABKWR138-07	–	USA	KP845421	–	–
139	CNCLEP00026530, [MIC6902♀]	MNAA372-07	–	Canada	KP845412	–	–
***Parectopa ononidis***							
140	CLV1785	GRSLO265-10	–	Austria	JN271915	–	–
141	CLV1797	GRSLO277-10	–	Austria	JF848374	–	–
142	CLV2269	GRSLO654-11	–	France	KP845416	–	–
143	CLV2272	GRSLO657-11	–	France	KP845388	–	–
144	CLV2283	GRPAL125-11	–	France	JN271901	–	–
145	CLV2284	GRPAL126-11	–	France	JN271902	–	–
146	F11onon	GRACI439-09	*Ononis* sp.	Hungary	KP845394	–	–
147	F12onon	GRACI440-09	*Ononis* sp.	Spain	KP845399	–	–
148	NK461	MICRU016-15	*Trifolium pratense*	Russia	KU380258	–	–
***Parectopa robiniella***							
149	CLV1860	GRSLO340-10	–	Italy	JF848420	–	–
150	CLV2282	GRPAL124-11	*Robinia* sp.	Slovakia	JN271900	–	–
151	CLV2542	GRPAL479-11	–	France	KP845390	–	–
152	CNCLEP00083021, [MIC6906♂]	MNAO1073-11	*Robinia pseudoacacia*	USA	KP845410	–	–
153	CNCLEP00083022, [MIC6973♂]	MNAO1074-11	*Robinia pseudoacacia*	USA	KP845392	–	–
154	CNCLEP00083023	MNAO1075-11	*Robinia pseudoacacia*	USA	KP845401	–	–
155	CNCLEP00083024	MNAO1076-11	*Robinia pseudoacacia*	USA	KP845409	–	–
156	CNCLEP00083025	MNAO1077-11	*Robinia pseudoacacia*	USA	KP845398	–	
157	FG58	GRPAL917-12	*Robinia pseudoacacia*	France	KP856956	–	

– no data.

### DNA sequence analysis

Sequence data for the barcode fragment (Hebert et al. 2003) were collected to estimate the barcode gap between *Micrurapteryx caraganella* and the related species. In addition, we sequenced two nuclear genes: histone H3 and 28S rDNA (28S) for *Micrurapteryx caraganella* and *Micrurapteryx gradatella* as an independent source of data to confirm the large divergence observed in the barcode fragment between these two species.

The primers used in both amplification and sequencing were LCO (5’ GGT CAA CAA ATC ATA AAG ATA TTG G 3’) and HCO (5’ TAA ACT TCA GGG TGA CCA AAA AAT CA 3’) for the COI gene (Folmer et al. 1994); H3 F (5’ ATG GCT CGT ACC AAG CAG ACG GC) and H3 R (5’ ATA TCC TTG GGC ATG ATG GTG AC) for the H3 gene (Colgan et al. 1998); and D1F (5’ ACC CGC TGA ATT TAA GCA TAT) and D3R (5’ TAG TTC ACC ATCTTT CGG GTC) for the 28S gene (Lopez-Vaamonde et al. 2001).

DNA from 22 specimens of *Micrurapteryx caraganella*, seven specimens of *Micrurapteryx gradatella* and one *Parectopa ononidis* was extracted, PCR amplified and sequenced at INRA (Orléans, France). DNA was extracted using NucleoSpin® tissue XS kit, Macherey-Nagel, Germany, according to the manufacturer’s protocol. The COI barcoding fragment, 658 bp, was amplified via PCR at the standard conditions for the reaction. PCR products were purified using the NucleoSpin® Gel and PCR Clean-up kit Macherey-Nagel, Germany and sequenced by the Sanger method with Abi Prism® Big Dye®Terminator 3.1cycle sequencing kit (25 cycles of 10 s at 96 °C, 5 s at 50 °C, 4 min at 60 °C). Sequencing was carried out using a 3500 ABI genetic analyzer. All sequences were aligned using CodonCode Aligner 3.7.1. (CodonCode Corporation).

DNA for the remaining samples was extracted and barcoded at the Canadian Centre for DNA Barcoding (CCDB, Biodiversity Institute of Ontario, University of Guelph) using the standard high-throughput protocol described in deWaard et al. (2008). In addition, 109 samples of North American species *Micrurapteryx salicifoliella*, *Micrurapteryx occulta*, and *Parectopa robiniella*, earlier barcoded by other colleagues, were also included in the analysis (Table 1).

The resultant sequences, along with the voucher data, images, and trace files, are deposited in the Barcode of Life Data Systems (BOLD) (Ratnasingham and Hebert 2007; www.barcodinglife.org) and the sequences were deposited in GenBank. All data are available through the following dataset (http://dx.doi.org/10.5883/DS-MICRURA)

Intra- and interspecific genetic distances were estimated using the Kimura 2-parameter model implemented within the analytical tools available in BOLD. We also used BOLD to obtain Barcode Index Numbers (BINs) (Ratnasingham and Hebert 2013). A neighbor-joining (NJ) tree was constructed using MEGA 5.05 (Tamura et al. 2011).

### Morphology

The external morphology of *Micrurapteryx caraganella* and the related species of *Micrurapteryx* and *Parectopa* was studied (Table 1, Suppl. material 1: Table S2). A total of 87 genitalia slides were examined (Table 1, Suppl. material 1: Table S2). Genitalia dissections and slide mounts prepared by PT (TRB slide numbers) and NK (NK slide numbers) followed Robinson (1976); those prepared by JFL (MIC, JFL, and USNM slide numbers) followed Landry (2007).

Genitalia imaged by PT were photographed with a Leica DFC 450 digital camera through Leitz Diaplan GMBH microscope. Those imaged by JFL were photographed with a Nikon DS-Fi1 digital camera mounted on a Nikon Eclipse 800 microscope at magnifications of 40× or 100× and Nikon’s NIS 2.3 Elements was used to assemble multiple images from successive focal planes into single deep-focus images. All photos and illustrations were processed, adjusted, and assembled into plates with Adobe Photoshop. Terminology of the genitalia follows Klots (1970) and Kristensen (2003); body larval chaetotaxy Kumata (1988), and that of the head Davis and Wagner (2005). Scanning electron microscope (ESEM) digital images of pupae were taken with a Hitachi TM1000.

Pinned specimens were photographed with a Canon EOS 60D with a MP-E 65 mm macro lens. They were placed on the tip of a thin plastazote wedge mounted on an insect pin, with the head facing toward the pin and the fringed parts of the wings facing outward. This ensured that there was nothing between the fringes and the background. Lighting was provided by a ring of 144 LEDs covered with a white diffuser dome (Fisher 2012 and references therein). The camera was attached to a re-purposed stereoscope fine-focusing rail. Sets of 30–65 images in thin focal planes were taken for each specimen and assembled into deep-focused images using Zerene Stacker, and edited in Adobe Photoshop.

### Specimen depositories

ANSP
Academy of Natural Sciences of Philadelphia, Philadelphia, Pennsylvania, U.S.A. 


BIO
Biodiversity Institute of Ontario, University of Guelph, Guelph, Ontario, Canada 


CNC
Canadian National Collection of Insects, Arachnids, and Nematodes, Agriculture and Agri-Food Canada, Ottawa, Ontario, Canada 


SIF
 Sukachev Institute of Forest, Siberian Branch of the Russian Academy of Sciences, Krasnoyarsk, Russia 


MSNV
Museo Civico di Storia Naturale, Verona, Italy 


USNM
 National Museum of Natural History, Smithsonian Institution, Washington, D.C., U.S.A. 


WSDA
Washington State Department of Agriculture, Olympia, Washington, U.S.A. 


## Results

### Molecular Analysis

#### DNA barcodes

**Figure 1.**
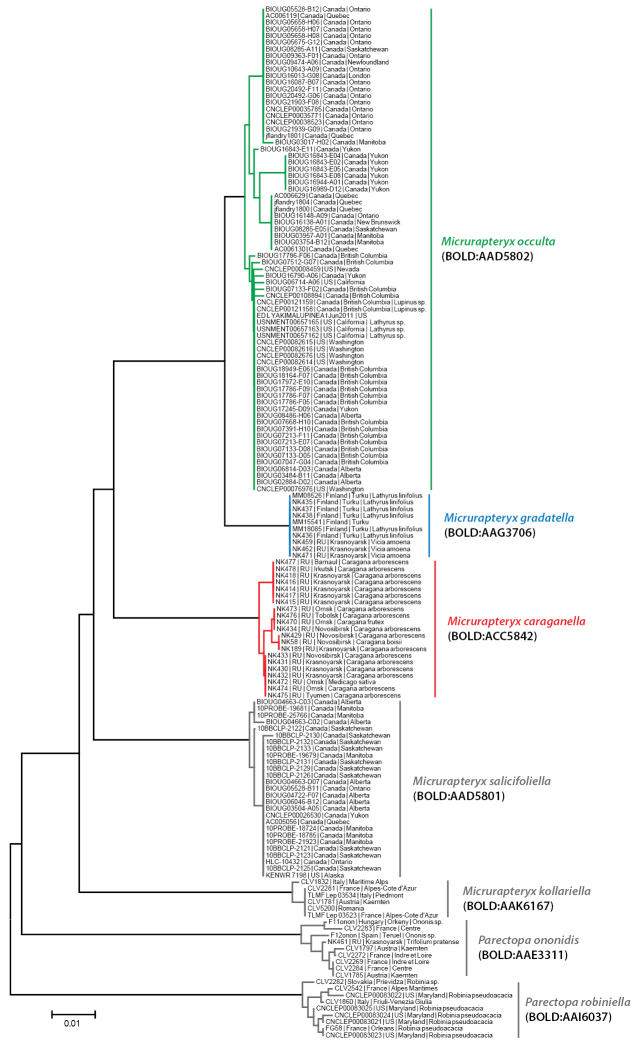
A Neighbor-Joining tree, based on COI barcode fragment, generated under the K2P nucleotide substitution model, of the studied taxa. Each specimen is identified by its Sample ID code (see Table 1). Branch lengths represent the number of substitutions per site. BIN numbers from BOLD system are given in parentheses for all clusters. There are 56 mutations and 9.2% interspecific distance between *Micrurapteryx caraganella* and *Micrurapteryx gradatella*.

In total, 157 DNA barcodes of specimens of the genera *Micrurapteryx* and *Parectopa* were analysed in this study: 22 – *Micrurapteryx caraganella*, 11 – *Micrurapteryx gradatella*, 73 – *Micrurapteryx occulta*, 6 – *Micrurapteryx kollariella*, 27 – *Micrurapteryx salicifoliella*, 9 – *Parectopa ononidis*, 9 – *Parectopa robiniella* (Table 1, Fig. 1). Barcoding of the two samples (*Micrurapteryx caraganella*, sample ID – NK439 and *Micrurapteryx gradatella*, sample ID – NK440) was not successful but their sequences with the genes 28S and histone H3 were obtained. There was a perfect correspondence between Barcode Index Numbers (BINs) membership and the known species (Fig. 1). The sequences of *Micrurapteryx caraganella* formed a distinct cluster (Fig. 1). We found 56 diagnostic substitutions in the barcode fragment between *Micrurapteryx caraganella* and *Micrurapteryx gradatella* (Suppl. material 1: Table S3). There is a clear barcode gap in the genus *Micrurapteryx* with a mean intraspecific divergence of 0.24% versus a nearest-neighbour (NN) distance averaging 5.84%. The lowest interspecific distance (2.0%) was observed between *Micrurapteryx gradatella* and specimens from North American *Micrurapteryx occulta* reared from *Lupinus* (Table 2). With DNA-barcoding, we identified *Parectopa ononidis* on *Trifolium pratense* in Siberia (Krasnoyarsk, Yenisei, Skala Berkut, 5.VII.2015, sample ID NK463) (Fig. 1), which is a new insect record for Siberia.

**Table 2.** Intra- and interspecific genetic divergences in DNA barcode sequences among studied species.

Species	*Micrurapteryx gradatella*	*Micrurapteryx caraganella*	*Micrurapteryx kollariella*	*Micrurapteryx salicifoliella*	*Micrurapteryx occulta*	*Parectopa ononidis*	*Parectopa robiniella*
***Micrurapteryx gradatella***	[**0.02**]						
***Micrurapteryx caraganella***	9.2	[**0.62**]					
***Micrurapteryx kollariella***	11.0	11.8	[**0.62**]				
***Micrurapteryx salicifoliella***	9.1	10.7	11.3	[**0.62**]			
***Micrurapteryx occulta***	1.9	7.7	10.3	8.0	[**1.66**]		
***Parectopa ononidis***	15.4	15.6	16.5	14.0	14.4	[**1.55**]	
***Parectopa robiniella***	16.2	16.2	16.2	14.6	14.3	14.1	[**1.1**]

Kimura 2-parameter (K2P) distances (%) for barcode DNA sequences of the eight analyzed species in the genera *Micrurapteryx* and *Parectopa*; minimal pairwise distances between species are given for each species pair; values in square brackets represent maximal intraspecific distances.

Within studied species, *Micrurapteryx gradatella* showed low intraspecific variability (0.02%) with ten specimens originating from one locality in Finland and one locality in Siberia (Table 2). All specimens from Finland, collected on *Lathyrus linifolius* shared the same haplotype. One mutation was observed in a Siberian specimen of *Micrurapteryx gradatella* (sample ID – NK459) sampled from a second host, i.e. *Vicia amoena*.

Intraspecific variability of *Micrurapteryx caraganella* reached 0.62% with 21 specimens collected from seven geographic locations throughout Siberia (Table 2). With DNA barcoding, *Micrurapteryx caraganella* was identified on the arborescent *Caragana* (*Caragana arborescens*, *Caragana frutex*, *Caragana boisii*) and on the herbaceous *Medicago sativa* (Fig. 1).

North American specimens of *Micrurapteryx occulta* formed a single large cluster belonging to one BIN (BOLD:AAD5802) which was nested close to *Micrurapteryx gradatella* within *Micrurapteryx*. Intraspecific variability at 1.66% was higher than for other species studied here but the geographic sampling was correspondingly much greater, covering 38 localities spanning the continent from East to West.

#### Nuclear genes

**Figure 2.**
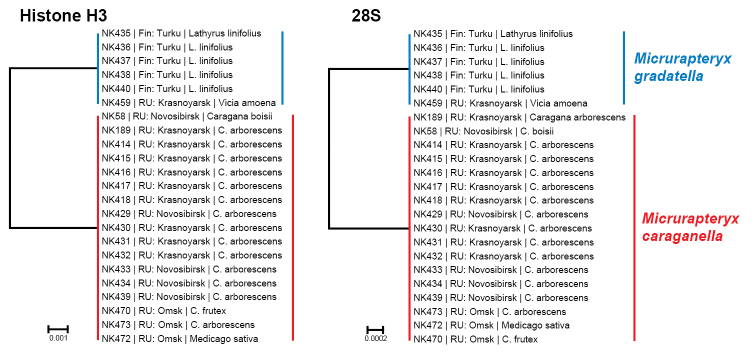
The Neighbor-joining trees, based on fragment of nuclear genes histone H3 and 28S, generated under the K2P nucleotide substitution model, of the studied taxa. Branch lengths represent genetic K2P divergences between the taxa according to the scale. Host plants are indicated for those specimens, which were bred from mines. Genetic divergence between *Micrurapteryx caraganella* and *Micrurapteryx gradatella* is due to three mutations in the histoneH3 gene (0.92% interspecific distance) and two mutations in the 28S gene (0.20 % interspecific distance).

We obtained sequences of the nuclear gene histone H3 and 28S rRNA D1-D3 for 23 specimens (17 specimens of *Micrurapteryx caraganella* and 6 specimens of *Micrurapteryx gradatella*, Table 1). Both H3 and 28S unequivocally delimit two distinct species with 3 and 2 diagnostic nucleotide substitutions respectively (Fig. 2; Suppl. material 1: Table S4). Sequencing these two genes confirm the presence of *Micrurapteryx caraganella* on both *Caragana* and *Medicago* in Siberia. No evidence of mitochondrial introgression between *Micrurapteryx caraganella* and *Micrurapteryx gradatella* was recorded.

### Morphology, biology, and distribution

Here the detailed morphological descriptions for three species are provided: *Micrurapteryx gradatella* (which has been confused with *Micrurapteryx caraganella* in the literature), *Micrurapteryx caraganella* and the closely related North American *Micrurapteryx occulta*.

#### Micrurapteryx gradatella

Taxon classificationAnimaliaLepidopteraGracillariidae

(Herrich-Schäffer, 1855)

Figs 3 , 13 , 18 , 24 , 25 , 40 , 41 , 59–64

##### Citations.

[No genus *Gradatella* Herrich-Schäffer, [1854]: plate 21: fig. 992 [unavailable]]

[*Euspilapteryx Gradatella* Herrich-Schäffer, [1855]: 293. Type locality: near Regensburg, Germany]

[*Gracilaria gradatella*; Staudinger and Rebel 1901: 208]

[*Parectopa gradatella*; Meyrick 1912: 21; Benander 1944: 122; Hering 1957: 600, 1110]

[*Micrurapteryx gradatella*; Spuler 1910: 409; Bengtsson and Johansson 2011: 103]

##### Original description.

*Alis anter. Margine interiore albo, triinciso. Etwas kleiner als vorige [kollariella], mit schmaleren Vorderflügeln, deren Vorderrandsstriche desshalb schräger stehen, aber feiner und länger sind, der erste geschlängelt, dem zweiten genähert, deren weisser Innenrund einwärts drei Zachen bildet, zwischen welchen die weisse Farbe tief schwarz ausgefüllt ist. Ich fand 3 Exemplare an verschiedenen Stellen bei Regensburg, im Mai*.

[English translation] “Somewhat smaller than previous, with narrower forewings, and front-marginal-dashes therefore more angled but finer and longer, the first sinuate [translates as ‘tortuous’], adjacent to the second, in which three inward teeth are formed by the white inner border, with deep black filling between the white colouration. I found 3 specimens in various places near Regensburg in May.”

##### Material examined.

Adult (9): 1♀, Norway, HEs, Elverum, Hernes, 1a, 28.VI.1981, *Lathyrus montanus*, O. Karsholt, slide TRB4060; 2♀, Norway, HEs, 20.VI.1961, Norway, *Lathyrus montanus*, K. Larsen, slide MIC6942; 1♂, Predota, Mezösig [Mezöseg, Cluj County, Romania], 24.6, slide TRB755; 2♂, FIN V [Finland], Turku, 670:23, e.l. 6.2000, T. Mutanen leg., *Lathyrus linifolius*, slide TRB4091, TRB4095; 1♂, FIN V [Finland], Turku, 670:23, e.l. 6.1998, *Lathyrus linifolius*, slide TRB4081; 2 ♂, Russia, Siberia, Krasnoyarsk (Yenisei river bank, near), *Vicia amoena*, 3.VII.2015, reared from mines, N. Kirichenko, slides NK-82-15-1, NK-82-15-2.

Pupa (7): Finland V: Turku, 6611:3230 mine, 12.6.2008 on *Lathyrus linifolius*, J. Itämies leg.; Finland V: Turku, 6714:234 mine, 19.06.2000 on *Lathyrus linifolius*, J. Itämies leg.; Finland, Ab Turku, collected June 2005 on *Lathyrus linifolius*, Markus J. Rantala leg. Larva (1): Finland, Ab Turku, collected June 2005 on *Lathyrus linifolius*, Markus J. Rantala leg.

##### Diagnosis.

Superficially, this species can be confused with *Micrurapteryx kollariella* (Figs 17, 20, 30–31, 47), widespread in Europe east to Kazakhstan. However, the latter can be distinguished by its forewing pattern with wider costal strigulae and white dorsal margin not denticulate. In male *Micrurapteryx kollariella*, the coremata are very long; the valvar apex is more protruded than in *Micrurapteryx gradatella*; the saccular apex has a strong, incurved bifurcate tooth; and the phallus is anteriorly widened and deeply invaginated and with fine lateral serrations (Figs 30, 31); in female *Micrurapteryx kollariella*, S6 is weakly sclerotized and less developed, the antrum is widest near the ostium, and the signa are a pair of finely denticulate plates (Fig. 47); in *Micrurapteryx gradatella* the antrum is elongate, cylindrical and widest more anteriorly. For differences with *Micrurapteryx caraganella*, see under that species.

##### Description of adult

**Figures 3–5.**
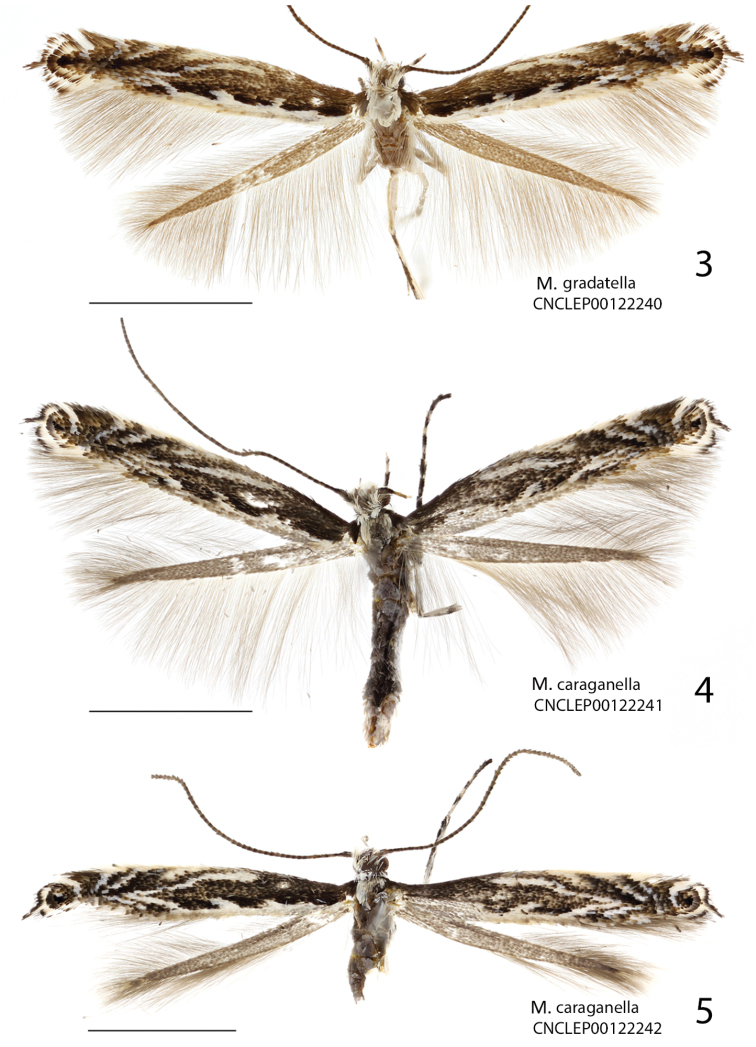
Adults of *Micrurapteryx* spp. **3** *Micrurapteryx gradatella*, specimen CNCLEP00122240 ♀ (Norway, Elverum) **4** *Micrurapteryx caraganella*, specimen CNCLEP00122241 ♀ (Russia, Krasnoyarsk) **5** *Micrurapteryx caraganella*, specimen CNCLEP00122242 ♀ (Russia, Krasnoyarsk). Scale bars: 2 mm.

(Fig. 3). Wingspan 9.5–11.5 mm.

*Head*. Frons and vertex white, sometimes with intermixture of brown scales on vertex, around eyes and at base of antenna. Labial palpus white, rather long and slender, upturned, spotted with dark brown in medial and apical segment; maxillary palpus about half of apical segment of labial palpus, outer side fuscous. Antenna fuscous, scape and pedicel white ventrally, remaining articles ringed with paler colour; pecten absent.

*Thorax*. Dorsum and venter white, tegulae dark brown. Legs white, tibiae and tarsi annulated with dark brown; fore coxa and femur grey outwardly. Forewing dark brown in ground colour with white markings; costal margin with 5 white strigulae; first three almost parallel, oblique and bent outwards; first costal strigula with basal half parallel to costa, then oblique and fragmented; second often obsolescent; fourth and fifth semicircular, often both touching opposite margin; dorsal margin white in basal two-thirds, with two or three white projections, the more distal one almost touching the first costal strigula; apical spot black, not quite touching 5^th^ strigula; cilia white around apex to tornus, with dark brown tips forming a line which projects a little at apex; hindwing grey ochreous, cilia pale grey.

*Abdomen*. Brown dorsally and white latero-ventrally. Segment 7 in the male with pair of coremata of thin scales about half width of sternum (Fig. 13). In the female sternum 6 more strongly sclerotized with a slight convexity on the proximal margin (Fig. 18).

*Male genitalia* (Figs 24, 25). Tegumen short, subtriangular, with no setae; tuba analis membraneous, braced by pair of sclerotized lateral bars, produced beyond tegumen, a small microspinose area ventroapically. Valva longitudinally cleft, costal margin slightly concave, cucullus lobe rounded; sacculus markedly developed, rectangular, lower margin with large, sharp, downward-oriented tooth, distal half lined with row of denticles. Phallus tubular, nearly as long as valva, straight, base bifurcate, dorso-medially with small spine, median ridge more or less serrated; vesica with two cornuti, first elongate, spear-like, one-third length of phallus, and second smaller, spiniform.

*Female genitalia* (Figs 40, 41). Anal papillae rather short, posterior apophyses shorter than anterior ones. Segment 8 short, about same length as anal papillae, weakly sclerotized. Sternum 7 markedly sclerotized, elongate-subtriangular. Ostium bursae rather narrow, rounded, at apex of S7. Antrum sclerotized, subcylindrical with anterior portion swollen; distal two-thirds of ductus bursae irregularly sclerotized with dense papillate microsculpture and one half-twist, proximal third membranous, inception of ductus seminalis ventrally on twisted portion. Bursa copulatrix slender, with pair of opposite signa each as cluster of 2–3 spines. Ductus spermathecae with efferent canal forming 3 or 4 coils before vesicle (not shown). Segment 6 shorter than or equal to preceding ones, sternum strongly sclerotized, transversely trapezoid, anterior margin with slight medial convexity.

##### Pupa.

Maximum length 5.5 mm; width 1.3 mm; vertex just shorter than frons. Frontal process (cocoon cutter) a transverse ridge strongly and irregularly dentate; frontal setae not visible, clypeal setae paired, very reduced and nearly contiguous. Antenna extended to abdominal segments A9; forewing to A5 or A6; hind leg to A10 or slightly longer than abdomen. Setae D1, L1 and SD1 present on abdominal segment A1-A7. Patočka and Turčáni (2005) report seta D1 on segment 7 but this was not found in the specimens examined. Cremaster consisting of a ring of five pairs of small spines, dorsal pair slightly enlarged and more closely set, two ventral pairs very small.

##### Larva.

Very similar to *Micrurapteryx kollariella and Micrurapteryx caraganella*. Last larval instars of this species were studied in detail by Grandi (1933) and no structural differences were discovered. For description, see *Micrurapteryx caraganella* below.

##### Biology.

*Lathyrus linifolius* (Reichard) Bässler [Syn. *Lathyrus montanus* Bernh., Lathyrus linifolius subsp. montanus (Bernhardi) Bässler, *Orobus tuberosus* L.], *Lathyrus tuberosus* L. and *Vicia sepium* L. (Hering 1957, Noreika 1997, De Prins and De Prins 2015, Bengtsson and Johansson 2011, Ellis 2015), *Lathyrus linifolius* in Finland (present study), *Vicia amoena* in Siberia (Figs 1, 59–61). Found in meadows and along forest edges. Flight period from mid-June to mid-July (Bengtsson and Johansson 2011). Larvae mine on the upper leaf surface, forming a blotch, initially whitish green then turning brown (Figs 59–62). Most frass is ejected from the mine (Hering 1957). Pupation takes place outside the mine (Figs 63–64).

##### Distribution.

*Micrurapteryx gradatella* is known from Finland, Norway, Sweden, Germany, Poland, Romania, Spain (Karsholt and Nieukerken 2015), Ukraine (Noreika 1997), Tajikistan (Puplesis et al. 1996), the central part of European Russia, the Urals, Siberia, and the Russian Far East (Amur oblast exclusively) (Sinev 2008). Reports from Tajikistan and the Urals need to be verified and, probably, those of the Russian Far East refer to *Micrurapteryx caraganella*.

#### Micrurapteryx caraganella

Taxon classificationAnimaliaLepidopteraGracillariidae

(Hering, 1957) comb. n.

Figs 4 , 5 , 14 , 19 , 26 , 27 , 42 , 43 , 49–54 , 55–58 , 65–76 , Suppl. material 4: S35, S36

##### Citations.

[*Parectopa* sp.; Hering 1957: 230]

[*Parectopa caraganella* Hering 1957: 1122. Type locality: Central Siberia]

[*Parectopa caraginella*; Dovnar-Zapol’skiy 1969: 36, subsequent incorrect spelling; Tomilova 1973: 8]

[*Gracilaria caraganella*; Dovnar-Zapol’skiy and Tomilova 1978: 34]

[*Micrurapteryx gradatella*; Kuznetzov 1981: 177, figs 173, 3–4; Kuznetzov and Tristan 1985: 189, figs 15–17; Noreika 1997: 380, figs 257–258; Kuznetzov and Baryshnikova 1998: 5–6; Kuznetzov 1999: 21, figs 3–4; misidentifications]

##### Material examined.

Adult (18): 1 ♂ *Caragana arborescens*, Krasnoyarsk, Akademgorodok, Yenisei bank 12.07.2013, N. Kirichenko, Kr-19-13-1, slide TRB3995♂; 1♀, 1 ex abdomen missing, *Caragana arborescens*, Krasnoyarsk, Akademgorodok, Yenisei bank 12.07.2013, N. Kirichenko, Kr-19-13-/2/4, TRB3986♀; 4 ♀, 1 ex abdomen missing, *Caragana arborescens*, Krasnoyarsk, Akademgorodok, Yenisei river bank, 18.08.2014, N. Kirichenko, slide TRB4061; 2 ♂, *Caragana arborescens*, Krasnoyarsk, Akademgorodok, Yenisei river bank, 18.08.2014, N. Kirichenko, slides MIC6940, MIC6941 (CNC); 1♂, 2♀, *Caragana arborescens*, Novosibirsk: SCBG SB RAS, 02.07.2013, N. Kirichenko, Nov-19-13-1/2/3, slide TRB3994♂, TRB4052♀; 2♀, *Caragana arborescens*, Krasnoyarsk, Akademgorodok, Yenisei bank, 15.07.2014, E. Akulov; 2 ♂, Russia, Siberia, Omsk (Victory park), *Caragana abrorescens*, 23.VII.2015, reared from mines, N. Kirichenko, slides NK-186-15-1, NK-186-15-2; 2 ♂, Russia, Siberia, Omsk (Victory park), *Caragana frutex*, 23.VII.2015, reared from mines, N. Kirichenko, slides NK-184-15-1, NK-184-15-2; 1 ♀, Russia, Siberia, Omsk (Victory park), *Caragana frutex*, 23.VII.2015, reared from mines, N. Kirichenko, slide NK-184-15.

Pupa (6): *Caragana arborescens*, *Micrurapteryx* sp., Russia, Krasnoyarsk, Akademgorodok, Yenisei river bank, 11.07.2013, N. Kirichenko, Kr-26-13. Larva (12): 5 larvae of the tissue-feeding instars, labelled as above, 12.07.2013, N. Kirichenko, Kr-19-13, 1 larva, *Caragana boisii*, Russia, Novosibirsk: SCBG SB RAS, 06.06.2012, N. Kirichenko, 22-12; 1 larva, *Caragana arborescens*, Russia, Novosibirsk: SCBG SB RAS, 03.08.2011, N. Kirichenko, Kr-30-11; 1 larva, *Caragana arborescens*, Russia, Omsk: Victory park, 23.VII.2015, N. Kirichenko, NK-186-15; 1 larva, *Caragana frutex*, Russia, Omsk: Victory park, 23.VII.2015, N. Kirichenko, NK-184-15; 1 larva, *Caragana arborescens*, Russia, Tyumen: Zatyumenskiy park, 24.VII.2015, N. Kirichenko, NK-209-15; 1 larva, *Caragana arborescens*, Russia, Tobolsk: Ermak garden, 25.VII.2015, N. Kirichenko, NK-212-15; 1 larva, *Caragana arborescens*, Russia, Barnaul: Izymrudniy park, 27.VII.2015, N. Kirichenko, NK-223-15.

##### Nomenclatural availability of *Parectopa caraganella* Hering, 1957.

The binomen *Parectopa caraganella* was first used by Hering (1957: 1122) who attributed it to Danilevsky without further indication. In his three-volume work, Hering (1957) distinguished the larva of a species of *Parectopa* from that of *Phytagromyza caraganae* E. Rodendorf (now *Aulagromyza caraganae* (Hering, 1957), see Ellis 2015) (Diptera, Agromyzidae), both being leaf miners on *Caragana* in Siberia. In his key on p 230 of volume 1, Hering wrote “Parectopa sp.” for species #1100a with the following “*Anfangsgang us. lang, epidermal. Kot im Platz teilweise ausgeworfen. Larva mit Kopfkapsel und Beinen … 1100a. Parectopa sp. (Lept.) Unterseite Gang seicht, weisslich. Oberseite Platz beginnt auf der Mittelrippe, kann das ganze Blättchen einnehmen, dieses und Mine gewechselt (Europa). 7,8 Central-Siberien (Buhr)*” (= “Beginning of mine on underside, long, epidermal. Frass partially ejected from mine. Larva with head capsule and legs … 1100a. Parectopa sp. (Lept.) Underside tunnel/gallery shallow, whitish. Upperside blotch begins on the midrib, can take the whole leaflet, this (e.g. the leaflet), and mine can be changed (Europe) 7,8. Central Siberia (Buhr).”). Thus Hering “described” the larva and its mine, albeit in an extremely minimalist way but sufficiently to distinguish it from the next taxon. The fact that the latter is a fly is irrelevant. Hering did not use the name *caraganella* on page 230. However, in volume 2 of the same publication (published simultaneously) on p 1122, in reference to volume 1, he listed a number of corrections. Thus page 1122 contains the following entry: “*p. 230, Nr. 1100a*: Parectopa caraganella *Danilevsky (statt* Parectopa *sp.)*” [“instead of *Parectopa* sp.”]. Again in the index on p 1164 Hering listed “Parectopa caraganella Danilevsky Suppl. 1100a”: the reference to entry #1100a undisputably links the taxon name to the description in the key of p 230.

Hering’s distinction in a key constitutes, however unintentionally, a valid description and thus makes the name *Parectopa caraganella* nomenclaturally available with Hering as the author. Despite being woefully inadequate, the “description” provided in Hering’s key minimally meets the criteria expressed in Article 13.1.1 of the Code, namely that a name published after 1930 (but before 1960) “be accompanied by a description of definition that states in words characters that are purported to differentiate the taxon” (International Commission on Zoological Nomenclature 1999).

It is worth noting that the description of the mine in association with the host plant provides a more useful diagnosis in the present case. Because the mine constitutes the work of an animal it could be construed as a condition for availability (Code article 12.2.8). However, such evidence is not admissible to assess the availability of names published after 1930.

Given its year of publication, a type specimen is not even required. Did Hering have voucher material of that species from Siberia when he wrote his 1957 work? He only mentioned the name “Buhr” at the end of the key couplet, who is presumably the person who communicated the information to him. He did not indicate how he obtained the name he attributed to Danilevsky. Even if so, the existence of voucher specimens would not affect the attribution of the name to Hering.

In a catalogue of leaf-mining insects, Dovnar-Zapol’skiy (1969) cited “*Parectopa caraginella* Dan.” (this seems to be a misspelling of *caraganella*) as a species feeding on *Caragana* described by Danilevsky from Western Siberia without any further reference or indication. As such, that citation has no nomenclatural value.

Kuznetzov and Tristan (1985) correctly discounted the names *Parectopa caraganella* Danilevsky and *Parectopa caraginella* Danilevsky as nomenclaturally unavailable. Indeed, despite being cited by several authors, no original publication by Danilevsky where either spelling of the name is mentioned seems to exist. It is intriguing that no authors who cited or attributed the names to Danilevsky gave any indication or reference where those names were seen in the first place.

##### Diagnosis.

The forewing pattern of *Micrurapteryx caraganella* is very similar to that of *Micrurapteryx gradatella* and the two species are separable with certainty only by examination of the genitalia. In male genitalia, *Micrurapteryx caraganella* differs mainly by the presence of a sharp, prominent tooth on the middle of the ventral margin of the valva. This character allows distinguishing easily this species from all other congeners. In female genitalia, the antrum is ampulla-shaped with lateral broadenings, whereas it is almost cylindrical in *Micrurapteryx gradatella*. The cremaster differs in pupae of the two species: there are three pairs of little spines in *Micrurapteryx gradatella* (Patočka and Turčáni 2005) versus five pairs in the new *Micrurapteryx caraganella*. The larva of *Micrurapteryx caraganella* differs modestly from those of *Micrurapteryx gradatella* and *Micrurapteryx kollariella* by the enlargements of the internal margins of the dorsal apodemes, along the epicranial notch.

##### Description of adult

(Figs 4, 5). Wing span 8.7–10.2 mm.

*Head*. Frons and vertex white, sometimes sprinkled with brownish grey. Palpi white; labial palpus rather long and slender, upturned, with apically forked dark brown band on median segment and sometimes apical one ringed with grey; maxillary palpus slightly more than half length of apical segment of labial palpus, spotted with fuscous outside. Antenna as in *Micrurapteryx gradatella*.

*Thorax*. Legs and thorax as in *Micrurapteryx gradatella*. Forewing dark brown in ground colour with white markings; costal margin with 5 white strigulae, the first four curving outwards, the fifth inwards, the first long and strongly oblique, the fourth often indistinct; dorsal margin with basal ⅔ white, this fascia denticulate inwards, often linked irregularly with costal strigulae; apical spot black with some mixture of paler scales, surrounded by circular white line including 5^th^ costal strigula; cilia and hindwing as in *Micrurapteryx gradatella*.

*Abdomen*. Brownish grey dorsally and white ventrally, apical segment with lateral dark grey spot in the female. Segment 7 of male similar to *Micrurapteryx gradatella*. Sternum 6 of female as in *gradatella* but posterior margin more rounded.

*Male genitalia* (Figs 26, 27). Tegumen short, triangular at apex, with no setae; tuba analis membraneous, without subscaphium, produced beyond tegumen, very similar to *Micrurapteryx gradatella*. Valva longitudinally cleft, costal region with sinuous margin, cucullus lobe rounded; sacculus with large, sharp tooth in middle of ventral margin and apex ventrally produced into strongly sclerotized toothed process with two pointed ends. Phallus tubular, about 0.9x length of valva, slightly bent in apical third, with small broadenings at base, a few small teeth on medio-ventral and dorsoapical walls and 2-3 larger denticles before apex; vesica with rather large patch of microspines and a thin, long cornutus apically pointed. Segment 7 with a pair of coremata of thin scales almost as long as width of sternum.

*Female genitalia* (Figs 42, 43). Anal papillae rather short, posterior apophyses shorter than anterior ones. Segment 8 about same length as anal papillae, weakly sclerotized. Sternum 7 markedly sclerotized, elongate-conical. Ostium bursae wide and rounded. Antrum sclerotized, ampulla-shaped, with lateral broadenings; inception of ductus seminalis near its anterior end; distal third of ductus bursae broadened, strongly and irregularly sclerotized with elongate-papillate microsculpture, medial third with thin lateral sclerotized band and proximal one completely membraneous. Bursa copulatrix slender, with pair of opposite signa each as cluster of 3–5 long spines. Ductus spermathecae with efferent canal forming 4 or 5 wide coils before vesicle (not shown). Segment 6 equal to preceding ones, sternum strongly sclerotized, posterior margin convexely rounded.

##### Pupa

(Figs 49–54). Maximum length 4.2 mm; width 0.9 mm. Head setae as in *Micrurapteryx gradatella*. Frontal process (cocoon cutter) a transverse ridge strongly and irregularly dentate. Antenna extended to abdominal segment A7, A8 or A10; forewing to A5, A6 or A7; hindleg from posterior margin of A7 to just beyond apex of abdomen. Setae D1, SD1 and L1 present on abdominal segment A1-7. Cremaster consisting of ring of five pairs of small recurved spines, two dorsal pairs slightly enlarged and more closely set, ventral pair very small.

##### Larva

(Figs 55–58). Tissue-feeding form examined of presumed last instar.

*Head*. Frons elongate, extended to epicranial notch, dorsal apodemes well developed, margins of epicranial notch with slight enlargement, on each side of caudal half while in *Micrurapteryx gradatella* these margins are regular; chaetotaxy with all three MD setae present, P2 very reduced; six stemmata on each side, arranged in 2 groups: first with 1 ventrad to A3, 2 between S2 and A3, 3, ventrad, near S2; second group in oblique line close to antenna. Mandible with 4 dorsal teeth and two ventral; both lateral setae present.

*Body*. Cuticle densely covered with very minute hairs, except on pronotal plate and small, symmetrical areas; chaetotaxy rather similar to that of *Acrocercops*-group (Kumata 1988): L setae bisetose on all segments except A9, SV bisetose on T1 and unisetose on T2-3, proprioceptor MD1 and MV3 present on T2-3 and A1-9; prolegs on A3-5 and A10. Most setae are inconspicuous, particularly the D and SV groups.

##### Biology

(Figs 65–76). The species usually mines the leaves of *Caragana arborescens* (Figs 65–69) but some individuals (i.e. larvae in mines) were also found on *Caragana frutex* (Figs 70–71), *Caragana boisii* (Fabaceae) (Figs 73, 74) and on the herbaceous *Medicago sativa* (Fabaceae) (Fig. 72). The mine is a roundish or slightly branched blotch (branches are short, 2-5 mm long) above the midrib (Figs 68–69). Often a long, narrow tunnel is visible on the lower surface of the leaf (Fig. 71). The mine quickly develops into an upper-surface flat blotch with digitate channels, occupying half or an entire leaflet (Figs 68, 69, 72, 73), similar to *Micrurapteryx gradatella* (Figs 59–62). Fresh mines are white (Figs 66–72) with larvae visible when examining the mines with backlighting (Figs 73). The larva consumes all layers of palisade parenchyma and partly damages the layers of spongy parenchyma. Since not all spongy parenchyma is eaten, the colour of the mine can be slightly greenish yellow. Larvae eject frass out of the mine by protruding the rear end of their body through a slit (up to 7 mm long) on the underside of leaves. Larvae can leave their mines (Fig. 74) and begin a new one, either on the same or a neighboring leaflet.

*Pupation* (Figs 75–76). Pupation takes place outside the mine, usually on the lower surface of a leaflet where the larva spins a transparent, glossy cocoon, locating it usually perpendicular to the midrib, as in case of *Micrurapteryx gradatella* (Figs 63–64). Silk deposition by the prepupa induces a slight buckle in the leaf so that presence of the cocoon can be detected from above by the curved appearance of the leaflet. Occasionally pupation may also occur on the upper side of a leaflet, at the base along the midrib (Fig. 75).

##### Phenology.

In Siberia, *Micrurapteryx caraganella* has two generations. The overwintering stage is not known (but is likely to be as a pupa or adult); neonate larvae of the first generation usually occur in early June. Adults fly in early July. The second generation develops from mid-July until the end of August.

##### Ecology and host plant range.

Leaf mines of the new species were most commonly found in Siberia on Siberian peashrub, *Caragana arborescens* (Fabaceae), a plant widely used for different purposes: as an ornamental, for erosion control, as a source of nectar for bees, and for nitrogen fixation (Shortt and Vamosi 2012). *Caragana arborescens* is native to Siberia, China, Mongolia, and Kazakhstan (Yingxin et al. 2010). In North America, where the shrub was introduced in 1752, it is naturalized and widespread (Shortt and Vamosi 2012).

Dovnar-Zapol’skiy and Tomilova (1978) mentioned *Vicia* sp. as a host plant for *Parectopa caraginella* / *Gracilaria caraganella*. Their record likely refers another *Micrurapteryx* species, particularly *Micrurapteryx gradatella* which is known to develop on *Vicia sepium* in Europe (Ellis 2015) and, according to our observations, on *Vicia amoena* in Siberia.

NK looked for mines of *Micrurapteryx* on *Vicia* spp. plants growing in the same locality as *Caragana arborescens* with mines of *Micrurapteryx caraganella*. No mines of *Micrurapteryx caraganella* were found on this herbaceous vetch, whereas leaf mines were common on *Caragana arborescens*. In Krasnoyarsk, on *Vicia*, particularly *Vicia amoena* NK recorded mines of *Micrurapteryx gradatella*.

These findings suggest that *Micrurapteryx caraganella* is an oligophagous insect with a preference for *Caragana arborescens*. In the Central Siberian garden SB RAS (Novosibirsk) in July 2012, NK also found a few mines of *Micrurapteryx caraganella* on *Caragana boisii*, an allied plant originating from China. In July 2015 in Omsk (Victory park), NK recorded mines of *Micrurapteryx caraganella* on *Caragana frutex* (native in Siberia). In the same location and at the same time bushes of *Caragana arborescens* were observed to be heavily attacked by *Micrurapteryx caraganella* (Figs 66, 67), whereas bushes of *Caragana frutex* growing in vicinity (20 m from the damaged *Caragana arborescens*) were hardly colonized by the insect. In Omsk, on the same plot, NK also found the occasional mines of *Micrurapteryx caraganella* on the herbaceous legume *Medicago sativa* growing near heavily infested Siberia peashrub *Caragana arborescens*.

##### Distribution.

Siberian regions previously considered part of the range of *Micrurapteryx gradatella*, namely Tyumen, Omsk, Kemerovo, Novosibirsk, Irkutsk oblats, Altai krai (Sinev 2008), the Republics of Buryatia and Yakutia (Sakha) (Dovnar-Zapol’skiy and Tomilova 1978), where it was recorded feeding on *Caragana*, most likely refer to *caraganella*. In July-August 2015, NK recorded *Micrurapteryx caraganella* at these locations, except in Kemerovo and Yakutia. Additionally, NK found it in the south of Krasnoyarsk krai and in the easternmost corner of Siberia, Transbaikal krai, in Chita (Victory Park). Also the reports of *Micrurapteryx gradatella* from Tajikistan and the Russian Far East (see above) probably belong to *Micrurapteryx caraganella*. There are no records of *Micrurapteryx caraganella* for North America where its host plant *Caragana arborescens* has been introduced as an ornamental.

#### Micrurapteryx occulta

Taxon classificationAnimaliaLepidopteraGracillariidae

(Braun, 1922) comb. n.

Figs 6–8 , 9–10 , 15 , 21 , 32–39 , 44–46

##### Citations.

[*Parectopa occulta* Braun, 1922: 91; McDunnough 1939: 98; Davis 1983: 9. Type locality: Powell County, Kentucky, U.S.A.]

[*Parectopa albicostella* Braun, 1925: 213; McDunnough 1939: 98; Davis 1983: 9; **syn. n.** Type locality: Spring Hollow, Cache County, Utah, U.S.A.]

##### Type material examined.

*Parectopa occulta*: Holotype female, in ANSP, labelled: “B. 1071, | Powell Co., | Ky. i. VII. 12. 21 [handwritten]; “TYPE | Collection of | Annette F. Braun” [red, printed]; “Parectopa | occulta | Type Braun” [handwritten with top and bottom black border]; “Specimen ID | CNCLEP | 00123636” [printed]; “genitalia slide | JFL 1748 ♀” [pale green, printed except sex symbol handwritten]. The “B. 1971” refers to a Braun rearing lot number and corresponding sheet of rearing notes preserved with her collection in ANSP. In the original description (Braun 1922) she provided the host information (*Vicia caroliniana* Walter) and observations on the larval mine and cocoon.

*Parectopa albicostella*: Holotype male, in ANSP, labelled: “B. 1199” [handwritten]; “Cache Co. Utah | i. VIII.5.24 | Annette F. Braun” [printed, second line handwritten]; “TYPE | Collection of | Annette F. Braun” [red, printed]; “Parectopa | albicostella | Type Braun” [handwritten with top and bottom black border]; “♂ genitalia on | slide 3764 | D.R. Davis” [printed with black border, number handwritten]; “Photograph | on file | USNM” [printed with blue border]; “Specimen ID | CNCLEP | 00123635” [printed]. Regarding the type locality, the holotype labels indicated only “Cache Co.” and no host but in her paper with the original description, Braun (1925) provided more precise information about the collecting site and indicated that it was reared from an undetermined “vetch” (presumably a herbaceous Fabaceae with *Vicia*-like foliage). The “B. 1199” refers to a Braun’s rearing lot number and corresponding sheet of rearing notes preserved with her collection in ANSP.

##### Other specimens examined.

See Tables 1, Suppl. material 1: Table S2.

##### Diagnosis.

Superficially, *Micrurapteryx occulta* is virtually indistinguishable from the other species treated here, especially when the substantial amount of individual variation in coloration is taken into account. Most specimens have the head, thorax, costal and dorsal margins and strigulae of the forewing white, contrasting sharply with the dark brown disk and ground color. However, in several specimens, the white areas are obscured by a suffusion of dark-tipped scales which gives them an overall dark, peppery appearance. The genitalia of both sexes are amply different from *Micrurapteryx salicifoliella*, the only other North American species (Figs 16, 22, 28, 29, 48). When compared to Palearctic *Micrurapteryx*, its genitalia are most similar to those of *Micrurapteryx gradatella*, from which it differs in having a single elongate cornutus and the latero-medial tooth projecting, whereas *Micrurapteryx gradatella* has a second cornutus consisting in a small, separate spine and its latero-medial tooth is elongate and flat. In the female genitalia of *Micrurapteryx occulta*, the posterior sclerotized papillate section of the ductus bursae is slightly shorter relative to the anterior membranous section, or less than half the length from the antrum to the corpus bursae; in *Micrurapteryx gradatella*, the papillate section extends to about two-thirds of the ductus length. The two species are closely related morphologically, genetically, and biologically.

##### Description of adult

**Figures 6–8.**
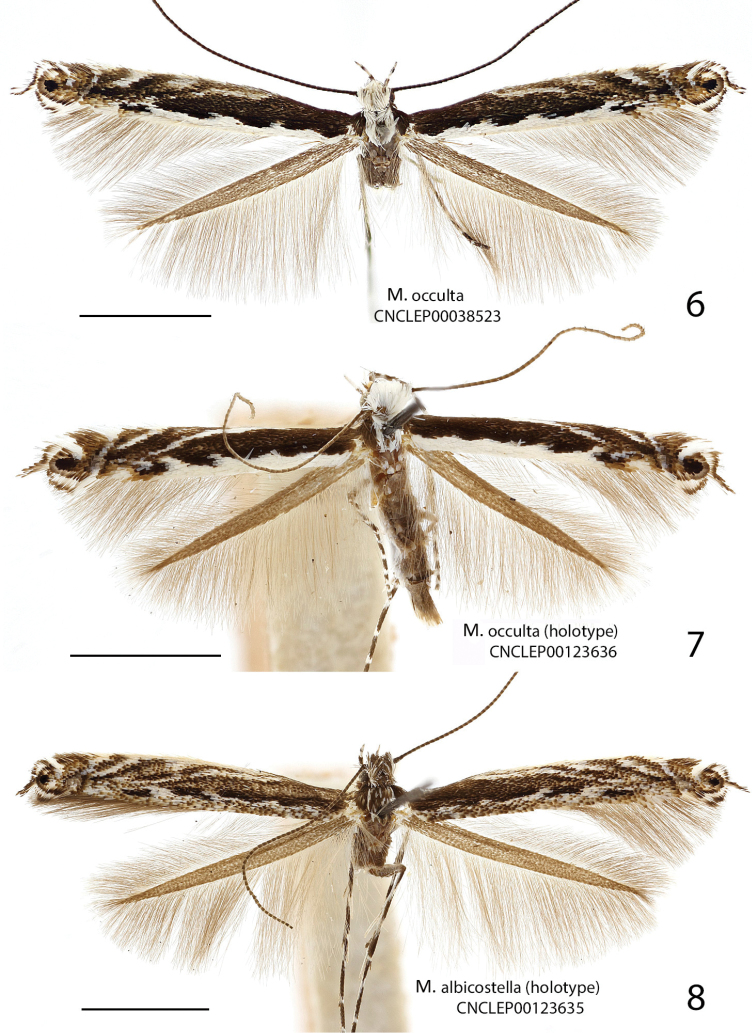
Adults of *Micrurapteryx*. **6** *Micrurapteryx occulta*, specimen CNCLEP00038523 ♂ (Canada, Ontario, Dunrobi) **7** *Micrurapteryx occulta* (“*Parectopa occulta*” holotype), specimen CNCLEP00123636 ♀ (USA, Kentucky, Powell County) **8** *Micrurapteryx albicostella* (“*Parectopa albicostella*” holotype), specimen CNCLEP00123635 ♂ (USA, Utah, Cache County, Spring Hollow). Scale bars: 2 mm.

**Figures 9–10.**
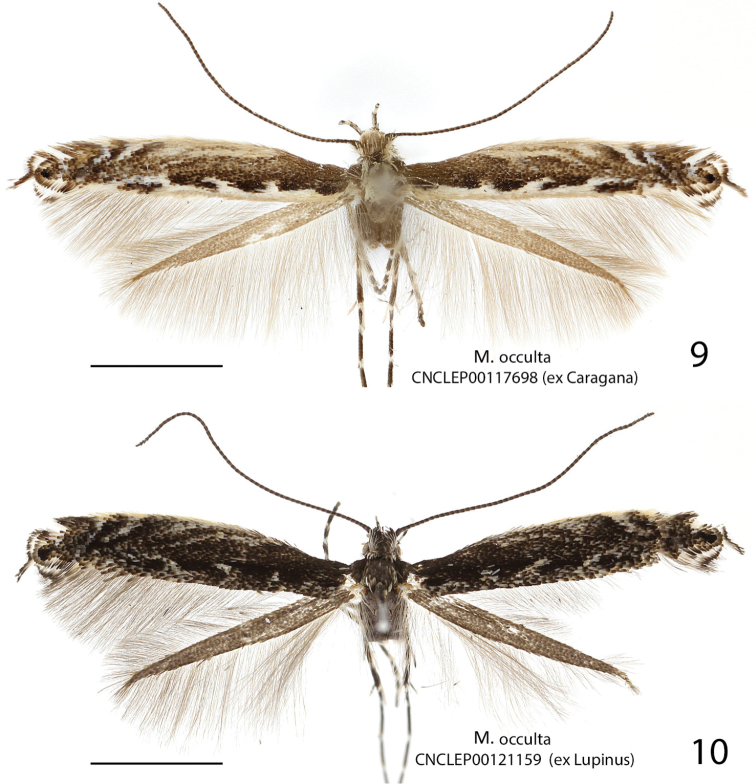
Adults of *Micrurapteryx*. **9** *Micrurapteryx occulta*, specimen CNCLEP00117698 ♀, ex *Caragana* (Canada, British Columbia, Lumby) **10** *Micrurapteryx occulta*, specimen CNCLEP00121159 ♂ ex *Lupinus* (Canada, British Columbia, Mt Kobau). Scale bars: 2 mm.

(Figs 6–10). Wingspan 8.7–11.7 mm (average 10.1 mm; 44 specimens).

*Head*. Frons and vertex white in most specimens, or dark from admixture of dark brown scales in dark specimens. Labial palpus shape as in *Micrurapteryx gradatella*, outer surface of article 2 dark brown, inner surface from all white to nearly all dark brown; article 3 variously ringed with dark brown in distal half in many. Antenna dorsally fuscous throughout, ventrally with scale, pedicel, and in many ¼ to ⅓ of flagellum white; pecten absent.

*Thorax*. Dorsum white in pale (most) specimens, predominantly dark brown peppered with white in dark specimens. Tegulae dark brown. Legs as in *Micrurapteryx gradatella*.

*Forewing*. Pattern very similar to that of *Micrurapteryx gradatella*, but rather variable: in several specimens, dark portion of disk with pale-based, dark-tipped scales giving the appearance of pale suffusion; white dorsal margin in some specimens obscured by suffusion of dark-tipped scales; terminal portion between strigulae 4 and 5 and around apical spot rufous in specimens with white costa and margin. Forewing of darker specimens with overall peppery appearance.

*Abdomen*. (Figs 15, 21). Pale grey dorsally, white ventrally. In male coremata of intersegmental membrane 6–7 about 0.5× width of S7.

*Male genitalia* (Figs 32–39, Suppl. material 2–4: Figs S01–S34). 32 preparations examined. Very similar to *Micrurapteryx gradatella*. Tegumen about 0.2× length of valva, with long and thin peduncular arms, apex subtriangular or subconical, with jagged edge, sometimes slightly indented. A pair of elongate lamellae about as long as tegumen-peduncular arms bracing the sides of anal tube, their distal portion with oblique wrinkles. Anal tube with 1 or 2 setae in few specimens, without seta in most. Latero-medial spine of phallus simple in most specimens, bitoothed in some specimens (including the holotype of *Parectopa albicostella*), tri-toothed observed in one specimen (Table 3), the spine projecting dorso-laterally from the phallus surface.

**Table 3.** Morphological variation in *Micrurapteryx occulta* from North America.

№	Specimen ID and genitalia preparation in []	BIN	Province / State	Head color	Thorax color	Forewing costa	Color of forewing apical area	Phallus		Anal tube setae	Signa
								median tooth	apical tooth		
1	AC006119, [MIC 6948♂]	BOLD:AAD5802	Québec	white	white	white	rufous	single	sharp	0	–
2	AC006130, [MIC 6939♂]	BOLD:AAD5802	Québec	white	white	white	rufous	single	sharp	2	–
3	AC006629, [MIC 6946♂]	BOLD:AAD5802	Québec	white	white	white	rufous	single	sharp	1	–
4	BIOUG02884-D02*, [MIC 7560♂]	BOLD:AAD5802	Alberta	–	–	–	–	single	blunt	0	–
5	BIOUG03017-H02*, [MIC 7553♂]	BOLD:AAD5802	Manitoba	–	–	–	–	single	sharp	0	–
6	BIOUG03484-B11*, [MIC 7458♂]	BOLD:AAD5802	Alberta	–	–	–	–	double sharp	sharp	0	–
7	BIOUG03754-B12*, [MIC 7556♀]	BOLD:AAD5802	Manitoba	–	–	–	–	–	–	–	7
8	BIOUG03957-A01*, [MIC 7557♀]	BOLD:AAD5802	Manitoba	–	–	–	–	–	–	–	4
9	BIOUG06714-A06*, [MIC 7455♂]	BOLD:AAD5802	California	–	–	–	–	double sharp	sharp	0	–
10	BIOUG06814-D03*, [MIC 7559♀]	BOLD:AAD5802	Alberta	–	–	–	–	–	–	–	4
11	BIOUG08285-A11*, [MIC 7555♀]	BOLD:AAD5802	Saskatchewan	–	–	–	–	–	–	–	8
12	BIOUG08285-E05*, [MIC 7460♀]	BOLD:AAD5802	Saskatchewan	–	–	–	–	–	–	–	5
13	BIOUG08486-H06*, [MIC 7561♂]	BOLD:AAD5802	Alberta	–	–	–	–	single	blunt	0	–
14	BIOUG09474-A06*, [MIC 7554♂]	BOLD:AAD5802	Newfoundland	–	–	–	–	single	sharp	0	–
15	BIOUG16138-A01*, [MIC 7457♂]	BOLD:AAD5802	New Brunswick	–	–	–	–	triple sharp	sharp double	0	–
16	BIOUG16843-E02*, [MIC 7456♂]	BOLD:AAD5802	Yukon	–	–	–	–	single	sharp	0	–
17	BIOUG16843-E05*, [MIC 7459♀]	BOLD:AAD5802	Yukon	–	–	–	–	–	–	–	4
18	BIOUG16843-E08*, [MIC 7558♂]	BOLD:AAD5802	Yukon	–	–	–	–	single	sharp	0	–
19	CNCLEP00007544, [MIC 6957♀]	barcode failed	Quebec	white	white	white	rufous	–	–	–	7
20	CNCLEP00008459, [MIC 6944♂]	BOLD:AAD5802	Nevada	white	white	white	pale brown	single	sharp	0	–
21	CNCLEP00016559, [MIC 6901♀]	BOLD:AAD5802	Quebec	white	white	white	rufous	–	–	–	2
22	CNCLEP00016560, [MIC 6955♀]	BOLD:AAD5802	Quebec	white	white	white	rufous	–	–	–	6
23	CNCLEP00016563, [MIC 6956♀]	BOLD:AAD5802	Quebec	white	white	white	rufous	–	–	–	4
24	CNCLEP00035771, [MIC 6945♂]	BOLD:AAD5802	Ontario	white	white	white	rufous	single	sharp	0	–
25	CNCLEP00035785, [MIC 6938♂]	BOLD:AAD5802	Ontario	white	white	white	rufous	single	sharp	0	–
26	CNCLEP00038523, [MIC 6839♂]	BOLD:AAD5802	Quebec	white	white	white	rufous	single	sharp	2	–
27	CNCLEP00076976, [MIC 6947♂]	BOLD:AAD5802	Washington	white	white	white	dark peppered	double sharp	sharp	0	–
28	CNCLEP00082614, [MIC 6943] ♂	BOLD:AAD5802	Washington	white	white	white	brown	single	sharp	0	–
29	CNCLEP00082615, [MIC 6953♂]	BOLD:AAD5802	Washington	white	white	white	brown	single	sharp	0	–
30	CNCLEP00082616, [MIC 6954♂]	BOLD:AAD5802	Washington	white	white	white	brown	single	sharp	0	–
31	CNCLEP00082676, [MIC 6937♂]	BOLD:AAD5802	Washington	white	white	dark peppered	dark brown	single	sharp small	0	–
32	CNCLEP00108894, [MIC 6949♂]	BOLD:AAD5802	British Columbia	white	white	white	dark peppered	single	sharp	0	–
33	CNCLEP00117698, [MIC 6903♀]	not barcoded	British Columbia	dark	white	white	brown peppered	–	–	–	5
34	CNCLEP00117700, [MIC 6966♀]	not barcoded	British Columbia	dark	dark	dark	dark peppered	–	–	–	6
35	CNCLEP00121158, [MIC 6904♀]	BOLD:AAD5802	British Columbia	white	white	white	dark peppered	–	–	–	6
36	CNCLEP00121159, [MIC 6905♂]	BOLD:AAD5802	British Columbia	dark peppered	dark peppered	dark peppered	dark peppered	double blunt	blunt	0	–
37	CNCLEP00123635, [DRD 3764♂] HOLOTYPE *albicostella*	not barcoded	Utah	dark	dark	white	brown peppered	double sharp	sharp small	0	–
38	CNCLEP00123636, [JFL 1748♀] HOLOTYPE *occulta*	not barcoded	Kentucky	white	white	white	rufous	–	–	–	5
39	CNCLEP00123677, [MIC 6950♂]	not barcoded	Quebec	white	white	white	rufous	single	sharp	0	–
40	CNCLEP00123684, [MIC 6951♂]	not barcoded	Quebec	white	white	white	rufous	single	sharp	0	–
41	CNCLEP00123694, [MIC 6958♀]	not barcoded	British Columbia	dark	dark	dark	dark peppered	–	–	–	3
42	CNCLEP00123994, [MIC 6963♀]	not barcoded	Manitoba	dark peppered	dark peppered	white	brown	–	–	–	4
43	CNCLEP00123996, [MIC 2151♂]	not barcoded	Manitoba	dark	white	white	brown peppered	single	sharp	0	–
44	CNCLEP00123997, [MIC 6962♂]	not barcoded	Manitoba	white	white	white	rufous	single	sharp	0	–
45	CNCLEP00124000, [MIC 6978♂]	not barcoded	British Columbia	dark	dark	dark peppered	brown	Single	Sharp	0	–
46	USNMENT00657161, [USNM 130245♀]	barcode failed	California	white	white	white	pale brown	–	–	–	4
47	USNMENT00657162, [USNM 130246♂]	BOLD:AAD5802	California	dark	dark	dark peppered	rufous	double sharp	sharp	0	–
48	USNMENT00657163 [USNM 130247♂]	BOLD:AAD5802	California	white	white	dark peppered	brown	double small	sharp	0	–
49	USNMENT00657165, [USNM 130248♂]	BOLD:AAD5802	California	dark peppered	dark peppered	dark peppered	pale brown	single	sharp	0	–

* malaise-trapped, ethanol-preserved.

– no data.

*Female genitalia* (Figs 44–46, Suppl. material 5–6: Figs S37–S52). 17 preparations examined. Very similar to *Micrurapteryx gradatella*. Sclerotized papillate section of ductus bursae about two-thirds length of ductus from antrum to corpus bursae. Number of spines of signa variable, 2–8 (average 5).

##### Notes about synonymy and variation.

The synonymy of *Parectopa albicostella* with *Micrurapteryx occulta* is here established based on examination of the type specimens of both nominal species. Braun described each species on the basis of a single specimen, which she reared. The holotype of *Micrurapteryx occulta* is a female reared from *Vicia caroliniana*, and that of *Parectopa albicostella* a male reared from an unspecified “vetch” (Fabaceae). We were not able to barcode the types. However, barcoded specimens of both sexes with genitalia corresponding to each of these nominal species cluster within a single, cohesive BIN (BOLD:AAD5802) comprised of specimens spanning a transcontinental geographic range. This cluster also includes specimens reared from different Fabaceae hosts that match the respective types in genital morphology and external appearance. Despite some morphological and genetic variation among examined specimens, we cannot find any consistent character to keep these two nominal taxa separate.

**Figures 11–12.**
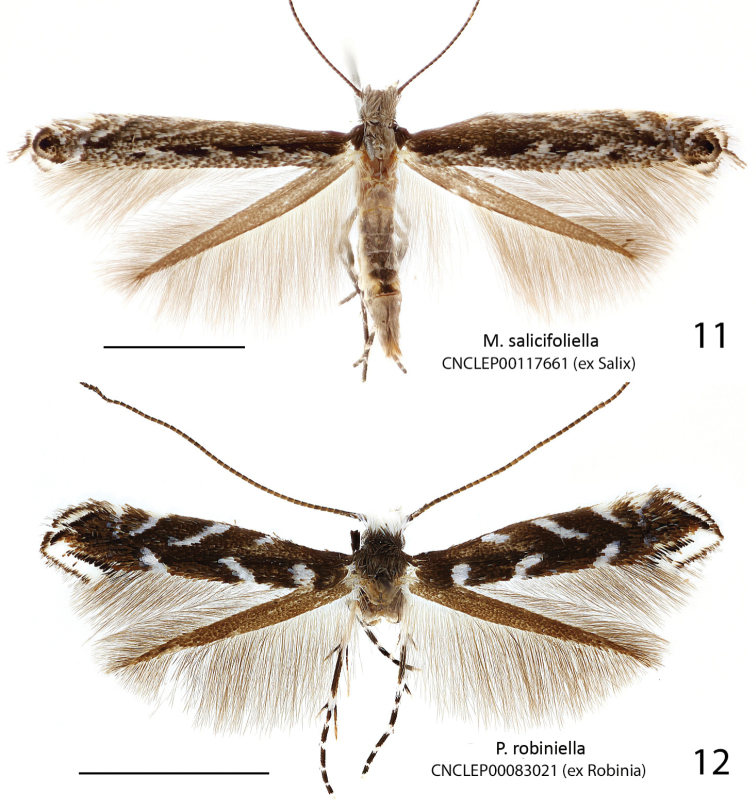
Adults of *Micrurapteryx* and *Parectopa* spp. **11** *Micrurapteryx salicifoliella*, specimen CNCLEP00117661 ♀ ex *Salix* (Canada, Ontario, Jellicoe) **12** *Parectopa robiniella*, specimen CNCLEP00083021 ♂ ex *Robinia* (USA, Maryland, Scientists Cliffs). Scale bars: 2 mm.

Braun (1925) indicated that *Micrurapteryx albicostella* was closely allied to *Micrurapteryx salicifoliella* Chambers (Fig. 11), *Parectopa thermopsella* Chambers, and *Micrurapteryx occulta* Braun, “but separated from all of them by the dark head and thorax and the white costal edge.” We observed that these colour characteristics vary individually among all specimens examined, including among *Micrurapteryx salicifoliella*. For example, a pair of *Micrurapteryx occulta* with identical barcodes reared from leafmines on the same lupine plant from British Columbia (specimens CNCLEP00121158 and CNCLEP00121159) shows the male with a dark head and thorax as well as a darkened dorsal edge as exhibited by the male holotype of *Parectopa albicostella*, whereas the female has a white head, thorax, and costal edge as in the female holotype of *Micrurapteryx occulta*. In fact, the holotype of *Parectopa albicostella* has the thorax predominantly dark peppered with white scales (Fig. 8, not really “streaked” as Braun described). Although this might suggest sexual dimorphism in colouration, both colour patterns (and others) were observed in each sex among the other specimens that we examined.

The genitalia of both Braun holotypes are not distinguishable from those of other barcoded specimens in BIN BOLD:AAD5802, as well as from several additional non-barcoded specimens examined. Although minor variations in several features were observed, these do not exhibit a clear geographic pattern (Table 3).

In male genitalia (32 preparations examined, Figs 32–39, Suppl. material 2–4: Figs S01–S34), for example, the lateromedial tooth of the phallus is simple in most specimens (Fig. 33) but double in a few western specimens (Figs 35, 39, including the *Micrurapteryx albicostella* holotype from Utah), with one from British Columbia showing a suggestion of blunt doubling, and even one eastern specimen from New Brunswick with a triple tooth (Fig. 37); the apical lobe of the sacculus is variously pointed or somewhat rounded (rounded in *Micrurapteryx albicostella* holotype from Kentucky); the curvature of the apex of cucullus varies from well rounded to nearly straight; and a single or a pair of fine setae are present on the membranous part of the anal tube in some specimens (Fig. 32). The anal seta character is uncommon in Gracillariinae – it may have been overlooked – and seems inconstant at the specific level. One seta is present in one male *Micrurapteryx kollariella* examined (Fig. 30).

In female genitalia (17 preparations examined), the number of signa varies from 2 to 8 (average 5), and the relative length and thickness of the antrum, sclerotized portion of the ductus bursae, and ostium notch vary slightly in proportions with no significant gap (Figs 44–46, Suppl. material 5–6: Figs S37–S52).

##### On “*Parectopa*” *thermopsella* (Chambers, 1875).

Braun (1922, 1925) also alluded to the relatedness of *Parectopa thermopsella* to *Micrurapteryx albicostella*, *Micrurapteryx occulta*, and *Micrurapteryx salicifoliella*, highlighting slight differences in forewing streaks, and this suggests superficially a similar external appearance and forewing pattern. It is not known whether Braun had seen authentic Chambers specimens of *Parectopa thermopsella*. Chambers (1875) mentioned his *Parectopa thermopsella* as “closely allied” to *Parectopa lespedezaefoliella* (type species of *Parectopa*), *Parectopa robiniella*, and *Micrurapteryx salicifoliella*, but his description of the larval mine immediately after that statement makes it unclear whether he was referring to the larval habits, the external appearance of the adult, or both. Both *Parectopa lespedezaefoliella* and *Parectopa robiniella* (Fig. 12) have forewing patterns unlike *Micrurapteryx* species but the larval mines are similar in appearance. The identity of *Gracilaria* [sic] *thermopsella* Chambers, 1875 remains unknown. The type locality is Spanish Bar, Colorado, and the host plant is a species of *Thermopsis* (Fabaceae). It has been included in *Parectopa* by subsequent authors (Braun 1925, McDunnough 1939, Davis 1983) but no type or other Chambers specimens seem to exist (Don Davis, pers. comm. to JFL, 2015).

##### Note on transferring *occulta* from *Parectopa* to *Micrurapteryx*.

Despite the long-standing combination of *occulta*/*albicostella* with *Parectopa*, DNA, the forewing pattern, and genitalia clearly indicate greater relatedness to members of *Micrurapteryx*.

##### Biology.

Recorded host plants include several Fabaceae, namely *Lathyrus japonicus* Willd. [Syn. *Lathyrus maritimus* (L.) Fr.] (Quebec), *Lathyrus* sp. (California), *Melilotus albus* Medik. (British Columbia, Manitoba, Ontario, Connecticut), *Vicia caroliniana* Walter (Kentucky, type of *occulta*), “vetch” (Utah, type of *albicostella*), *Lupinus* sp. (British Columbia), *Caragana* sp. (British Columbia). It was collected in meadows, at the edge of forests, in open ponderosa pine forests (Washington), in alpine meadows (British Columbia), along the sea shore (Quebec), and probably other habitats, from sea level to high elevations in the mountains (Nevada), where suitable hosts occur. Records indicate two generations, at least over parts of its range, with most adult records in mid-summer. Early seasonal records in March – April as well as late-flying adults in October – December found indoors in southern Canada (Quebec, Ontario) suggest overwintering in the adult stage.

##### Distribution.

*Micrurapteryx occulta* is here recorded from across North America in the northern half of the continent, in Canada from the Maritime Provinces (Newfoundland, New Brunswick, Nova Scotia) to British Columbia, north to northernmost Yukon; in the United States it has been found in Connecticut (D.L. Wagner, pers. comm.), Kentucky, Illinois (T. Harrison, pers. comm.), Colorado (E. van Nieukerken, pers. comm.), Utah, Nevada, and California.

## Discussion

**DNA barcoding and the status of *Micrurapteryx* species.** Siberia has a rich fauna of Lepidoptera which is still very poorly documented (Sinev 2013). So far, about 50 species of Gracillariidae are known to occur in Siberia on woody plants (Tomilova 1973; Dovnar-Zapol’skiy and Tomilova 1978; Kuznetzov and Baryshnikova 1998; Sinev 2008) but most of the region remains unexplored. Here, we confirm the existence of a distinct species of *Micrurapteryx*, namely *Micrurapteryx caraganella* feeding on plants from the genus *Caragana* (mainly on Siberian peashrub *Caragana arborescens*) and occasionally on *Medicago sativa* (Fabaceae) in Siberia based initially on differences in DNA barcodes. The status of *Micrurapteryx caraganella* is also supported by nuclear data, male and female genital morphology and biology.

In a review of Palearctic *Micrurapteryx* by Kuznetzov and Tristan (1985) considered that the *Caragana*-feeding *Micrurapteryx* present in Siberia were all referable to *Micrurapteryx gradatella*. However, it is clear from their description and illustrations of that species that it is markedly different in male and female genitalia from what is regarded as *Micrurapteryx gradatella* in Europe. Instead, their *Micrurapteryx gradatella* corresponds to our concept of *Micrurapteryx caraganella*.

In North America DNA barcodes revealed that a single species with a wide continental distribution is present, but that a significant amount of morphological variation was found among numerous specimens, supporting the synonymy of two long-standing nominal species, *Parectopa albicostella* and *Parectopa occulta*. Barcodes and morphology also supported the transfer of *Parectopa occulta* to *Micrurapteryx*.

The average interspecific divergence for the DNA barcode fragment found within *Micrurapteryx* (11.5%) is similar to other Gracillariidae such as *Cameraria* and *Phyllonorycter* (Langmaid et al. 2011). The relatively high level of DNA barcode divergence found between *Micrurapteryx caraganella* and *Micrurapteryx gradatella* contrasts with the limited differentiation in the two nuclear genes sequenced (i.e. H3 and 28S) (Table 2). The striking difference in the level of divergence between mitochondrial and nuclear genes could be caused by maternally inherited symbionts such as *Wolbachia* (Kodandaramaiah et al. 2013). A study on *Wolbachia* infection of both species is needed to confirm the role of this endosymbiont on the levels of mitochondrial and nucleotide diversity observed.

**Host range in *Micrurapteryx*.** The genus *Micrurapteryx* comprises species feeding on more than twenty different genera of legumes, and a host shift from Fabaceae to Salicaceae (Suppl. material 1: Table S1). In North America *Micrurapteryx occulta* has been recorded on several different genera of Fabaceae hosts (*Caragana*, *Lathyrus*, *Lupinus*, *Melilotus*, *Vicia*) (see specimens examined in DS-MICRURA dataset and Suppl. material 1: Tables S1, S2). Historic records of *Parectopa thermopsella* (reared from *Thermopsis* in Colorado) may also be referable to this species (although no authentic specimens of this nominal species are known). However, most individual *Micrurapteryx* species are specialized on one or two host plant genera. Our findings add more evidence to the prevalence of relatively high levels of host plant specialization. *Micrurapteryx sophorivora* Kuznetzov & Tristan, 1985 is restricted to *Sophora* sp. *Micrurapteryx gradatella* is known to feed only on *Lathyrus* and *Vicia* and is found in North Europe exclusively on *Lathyrus linifolius*. In Siberia, *Micrurapteryx caraganella* can occasionally colonize other *Caragana* species, besides *Caragana arborescens*, for example *Caragana frutex* and *Caragana boisii*. The species is also able to develop on the herbaceous legume *Medicago sativa*. Such a host shift from a woody shrub to a herbaceous plant is uncommon in Gracillariidae, which typically have strict diets and where occasional host shift usually do not take place between structurally different plant species. We recorded a new host genus (*Medicago*) only in one location in Siberia (Omsk) where *Micrurapteryx caraganella* was highly abundant and was severely defoliating *Caragana arborescens*, and thus could disperse to a nearby herb. It is possible that *Medicago* does not represent a normal food plant for *Micrurapteryx caraganella*, but that its occurrence on that host resulted from a local mass-occurrence and a consequent “spill-over effect”. Such a phenomenon is reported in other leaf miner species, including the horse-chestnut leafminer *Cameraria ohridella* Deschka & Dimić, 1986, a recent invasive pest of horse chestnuts (*Aesculus hippocastanum* L.) in Europe (Šefrova and Laštuvka 2001). Along with outbreaks and co-presence of the related maples (*Acer* spp.), mines of the horse-chestnut leafminer can be found on maples, although in lower abundance (Gregor et al. 1998; Péré et al. 2010). Similarly, *Ectoedemia occultella* (Linnaeus, 1767) (Nepticulidae), an abundant leaf miner of birch trees (*Betula* spp.), has been once reported to feed on an unrelated willow *Salix pentandra* L. (Johansson et al. 1990). In other gracillarid species, we have occasionally observed atypical host shifts, and these events often are associated with elevated population numbers, e.g. in *Phyllonorycter hilarella* (Zetterstedt, 1839) (from *Salix* spp. to *Populus tremula* L., observations by MM, see also Bengtsson and Johansson 2011); *Parectopa sorbi* (Frey, 1855) (observations verified by barcoding by MM from *Sorbus* spp. to *Prunus padus* L., *Parectopa domestica* L., *Parectopa avium* L., *Malus* spp. and *Crataegus* spp.; also a record verified by barcoding on *Chaenomeles* sp. (C. Doorenweerd, in litt.)); and *Phyllocnistis labyrinthella* (Bjerkander, 1790) (from *Populus* spp. to *Salix pentandra*, observations by MM). We observed significantly lower abundance of *Micrurapteryx caraganella* mines on *Medicago*, which we consider supporting the spill-over hypothesis, but on the other hand, we have not monitored the presence of mines on *Medicago* over several years, and therefore cannot exclude the possibility that it is part of the normal diet of *Micrurapteryx caraganella*.

**Differential diagnoses of *Micrurapteryx* and *Parectopa*.** The original descriptions of these two genera (for *Parectopa*: Clemens 1860: 209; for *Micrurapteryx*: Spuler 1910: 409) focused exclusively on external features of the head, antennae, palpi, wing shape, and venation, as was customary at that time.

Spuler (1910: 409) defined *Micrurapteryx* on account of the apex of the forewing being tail-like (hence the name): this appearance results from a thin “pencil” or line of dark fringe scales at the apex of the forewing which stand out from the surrounding white fringe scales, and thus make the wing appear “tailed”. This appearance is further accentuated by a rim of white scales between the apical dark spot and the base of the “tail”. In *Parectopa*, there is also a thin line of dark fringe scales at the apex of forewing but the dark outer edge of the fringe surrounds it so that it does not look “tailed”.

In describing *Parectopa*, Clemens (1860: 209) presented the description of the forewing venation first, emphasizing (italics in his text) the lack of “costal nervure” (Sc?) and the “three-branched” median vein (instead of four, as when CuA1 and CuA2 are both present, meaning these two veins are coincident or fused).

Vári (1961) cited verbatim the original descriptions of both genera and added genitalia characters as well as venational and leg details, but his re-descriptions do not provide clear distinctions between the genera other than for venation. In his treatment of *Micrurapteryx* he stated “Probably allied to *Parectopa*, but differing from it by [forewing] veins 2 (CuA2) and 3 (CuA1) being stalked and the male genitalia” (Vári 1961: 55), as opposed to being coincident in *Parectopa*. This venational feature is indicated by Spuler (1910: 409, legend of fig. 160) to be variable in *Micrurapteryx*. The value of these minor venational differences has not been assessed. The genitalia characters as provided by Vári are not easily comparable between the two genera (see Suppl. material 1: Table S5). Despite indicating that he “greatly restricted” *Parectopa* and reinstated *Micrurapteryx* as valid, Vári did not list which species he examined for both genera, although it can be assumed from his discussion that these included at least the type species.

In addition to DNA barcodes that cluster species into different sets of BINs and segregated *Micrurapteryx* from *Parectopa*, we noted several morphological characters not formulated by previous authors that distinguish the two genera from each other (Figs 77–86). These character states are likely mixtures of apomorphies and plesiomorphies. Without a phylogenetic framework for the genera of Gracillariinae and a more comprehensive mapping of characters across genera, it remains premature to assign character polarities and apomorphies that would support either the monophyly of each genus, or whether *Micrurapteryx* and *Parectopa* form a single monophyletic clade and should be combined. However, the differences are compelling enough to support the proposed new combinations. Provisional diagnoses for each genus follow. The characters presented are not meant to be exhaustive. We focused on abdominal and genital characters, and did not examine wing venation nor other skeletal features. We did not conduct a comprehensive survey of all the species currently attributed to each genus. However, the character states given here were present in all those examined (listed in Table 1 and Suppl. material 1: Table S2).

Character states shared by the examined species of *Micrurapteryx*:

*Forewing* with pattern of long, oblique costal streaks, broad, white dorsal margin, distinct dark apical spot between last costal strigula and fringe; apical fringe with thin line of dark scales extended from the apical spot and making the wing appear “tailed” (Figs 3–11).

*Male abdomen* with S1–2 venulae regularly incurved and apically without apodemes projected beyond anterior margin of sternum (Fig. 77). T7 with small, elongate-conical sclerotized area and indistinctly thickened anterior margin. S7 weakly sclerotized, unmargined. Intersegmental membrane 6–7 with pair of densely packed coremata of relatively short (less than width of abdominal segment) scales. T8 reduced to thin, narrow transverse band, without specialized scales. S8 reduced, weakly sclerotized. Pleura 8 without coremata. (Fig. 79).

*Female abdomen* with S1–2 similar to male. S6 sclerotized, transverse, markedly distinct from other sterna (Figs 18–22).

*Male genitalia* (Fig. 81) with vinculum broad, saccus area proportionally large. Pedunculi of tegumen as thin, simple arms, distal portion of tegumen distinctly delineated, subtriangular or conical. Phallus base with pair of posteriorly oriented “winglets”, outer wall of shaft ornate with spines, dorsally or ventrally, singly or in rows, and an elongate, spear-shaped cornutus (a second, small separate cornutus in some).

*Female genitalia* (Figs 83, 85): ductus bursae sclerotized over ½ of its length, sclerotized portion with papillate microsculpture. Signa present, either as pair of clusters of thorn-like spines (varying number) or scobinate patches.

Contrastingly, character states shared by the examined species of *Parectopa*:

*Forewing* with pattern of short costal and dorsal streaks, dorsal margin concolorous with disk, apical spot absent (Fig. 12).

*Male abdomen* with S1–2 venulae sinuate and anteriorly extended into free apodemes projected beyond anterior margin of sternum (Figs 23, 78). T7 well sclerotized, transverse, anteriorly margined with antero-lateral corners prolonged into tapered strut which abuts similar structure of S7. S7 sclerotized with thickened anterior margin. Intersegmental membrane 6–7 with pair of very long coremata (longer than width of abdominal segment). T8 elongate-conical with posterior margin lined with dense row of flatly broadened scales. S8 completely membranous, reduced, indistinct. Pleura 8 with pair of elongate coremata with scales in transverse, fan-like arrangement (Fig. 80).

**Figures 13–23.**
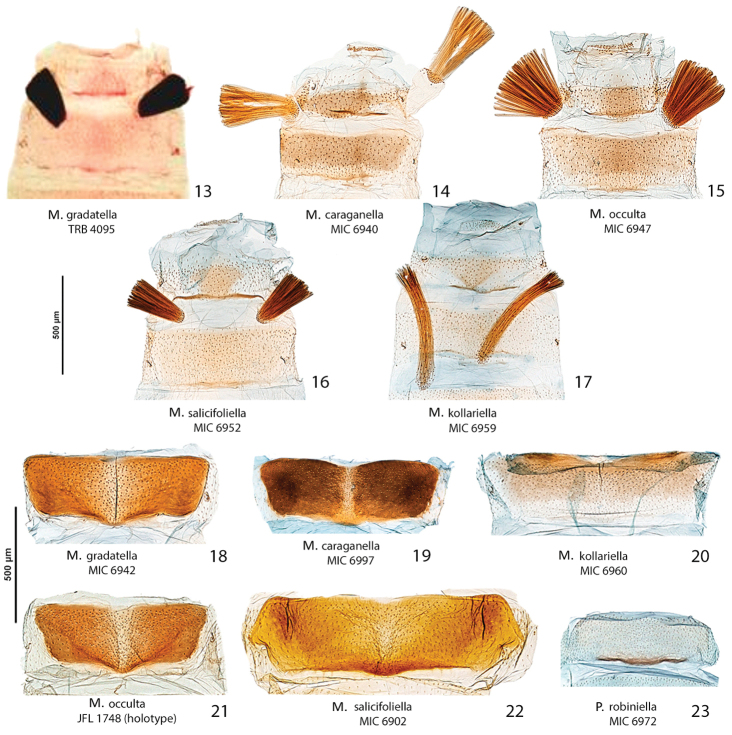
Male and female abdomens of *Micrurapteryx* and *Parectopa* spp. For males, segments 6–8 is shown; for females, sternum 6 is shown; posterior end oriented upward. **13** *Micrurapteryx gradatella* ♂ (slide TRB4095) (Finland, Turku) **14** *Micrurapteryx caraganella* ♂ (slide MIC6940, specimen CNCLEP00122241) (Russia, Krasnoyarsk) **15** *Micrurapteryx occulta* ♂ (slide MIC6947, specimen CNCLEP00076976) (USA, Washington) **16** *Micrurapteryx salicifoliella* ♂ (slide MIC6952, specimen CNCLEP00123690) (Canada, Ontario, Manitoulin Island) **17** *Micrurapteryx kollariella* ♂ (slide MIC6959, specimen CNCLEP00123697) (Germany, Berlin) **18** *Micrurapteryx gradatella* ♀ (slide MIC6942, specimen CNCLEP00122240) (Norway, Norvegica) **19** *Micrurapteryx caraganella* ♀ (slide MIC6997, specimen CNCLEP00132306) (Russia, Omsk) **20** *Micrurapteryx kollariella* ♀ (slide MIC6960, specimen CNCLEP00123698) (Germany, Berlin) **21** *Micrurapteryx occulta* ♀ holotype (slide JFL1748, specimen CNCLEP00123636) (USA, Kentucky) **22** *Micrurapteryx salicifoliella* ♀ (slide MIC6902, specimen CNCLEP00026530) (Canada, Yukon) **23** *Parectopa robiniella* ♀ (slide MIC6972, specimen CNCLEP00132251) (Canada, Nova Scotia, Smiths Cove). Scale bars: 500 µm.

*Female abdomen* with S1–2 similar to male but venulae straight. S6 weakly sclerotized, not markedly distinct from other sterna.

*Male genitalia* (Fig. 82) with vinculum elongate-narrow, saccus area proportionally very small. Pedunculi of tegumen with transparent “window” between base of valval costa and tuba analis, distal portion of tegumen indistinctly delineated. Phallus without spines nor cornuti, with apex attenuated into thin dorsally-oriented, acuminate point.

*Female genitalia* (Figs 84, 86): antrum short, less than ⅓ length of S7. Ductus bursae sclerotized over 3/4 to 4/5 of its length, sclerotized section mostly smooth except one area covered with very fine, slender spinules. Signa absent.

In conclusion, our study documents another example of how DNA barcoding can help to reveal overlooked species and clarify taxonomic issues (Jin et al. 2013; Landry et al. 2013; Lees et al. 2013; Mutanen et al. 2013; Huemer et al. 2014). Moreover, our analysis highlights the need for a careful revision of *Parectopa* and *Micrurapteryx* in the Nearctic and Palearctic Regions, particularly in the context of a broader phylogenetic analysis of the Gracillariidae.

**Figures 24–31.**
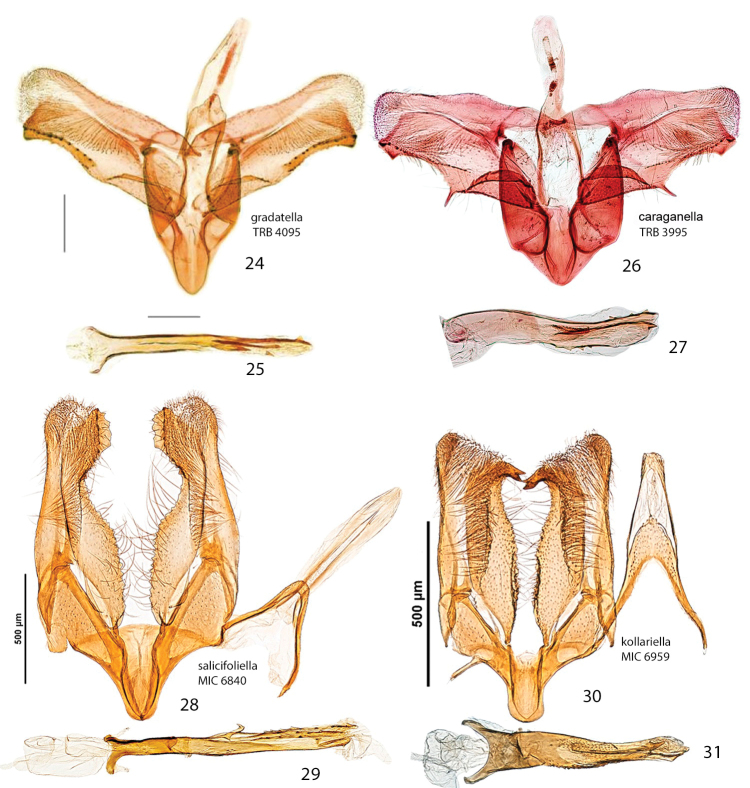
Male genitalia and phallus of *Micrurapteryx*.**24–25** *Micrurapteryx gradatella* (slide TRB4095) (Finland, Turku) **26–27** *Micrurapteryx caraganella* (slide TRB3995) (Russia, Krasnoyarsk) **28–29** *Micrurapteryx salicifoliella* (slide MIC6840, specimen AC005056) (Canada, Quebec) **30–31** *Micrurapteryx kollariella* (slide MIC6959, specimen CNCLEP00123697) (Germany, Berlin). Scale bars: 200 µm (**24, 26**), 250 µm (**25, 27**), 500 µm (**28–31**).

**Figures 32–39.**
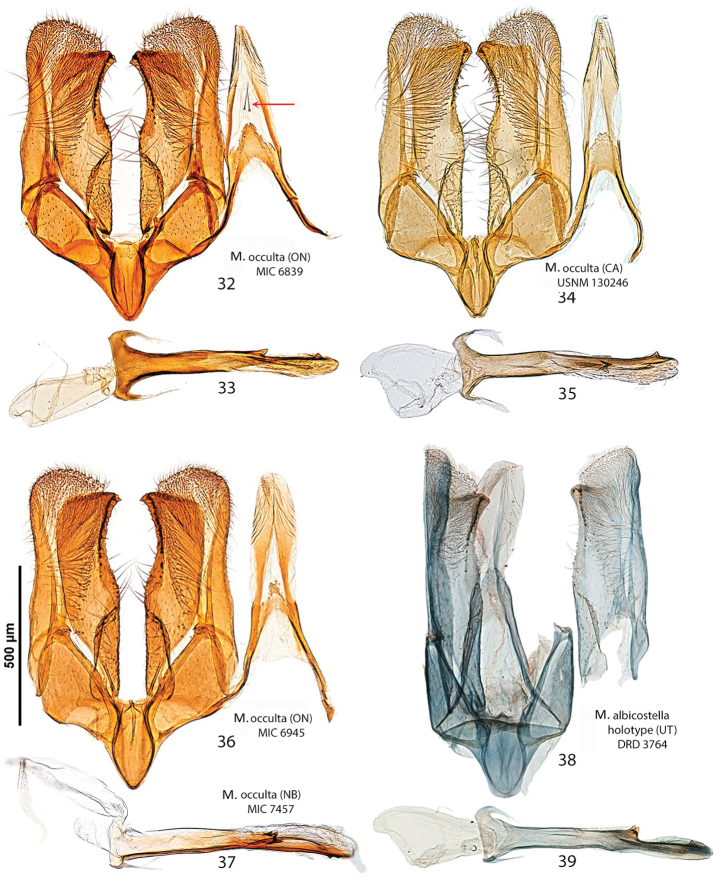
Male genitalia and phallus of *Micrurapteryx*. **32–33** *Micrurapteryx occulta* (slide MIC6839, specimen CNCLEP00038523) (Canada, Ontario) **34–35** *Micrurapteryx occulta* (slide USNM130246, specimen USNMENT00657162) (USA, California) **36** *Micrurapteryx occulta* genitalia (slide MIC6945, specimen CNCLEP00038523) (Canada, Ontario) **37** *Micrurapteryx occulta* phallus (slide MIC7457, specimen BIOUG16138-A01) (Canada, New Brunswick); note triple medial tooth **38–39** *Micrurapteryx albicostella* (“*Parectopa albicostella*”) holotype (slide DRD3764, specimen CNCLEP00123635) (USA, Utah). Scale bars: 500 µm.

**Figures 40–43.**
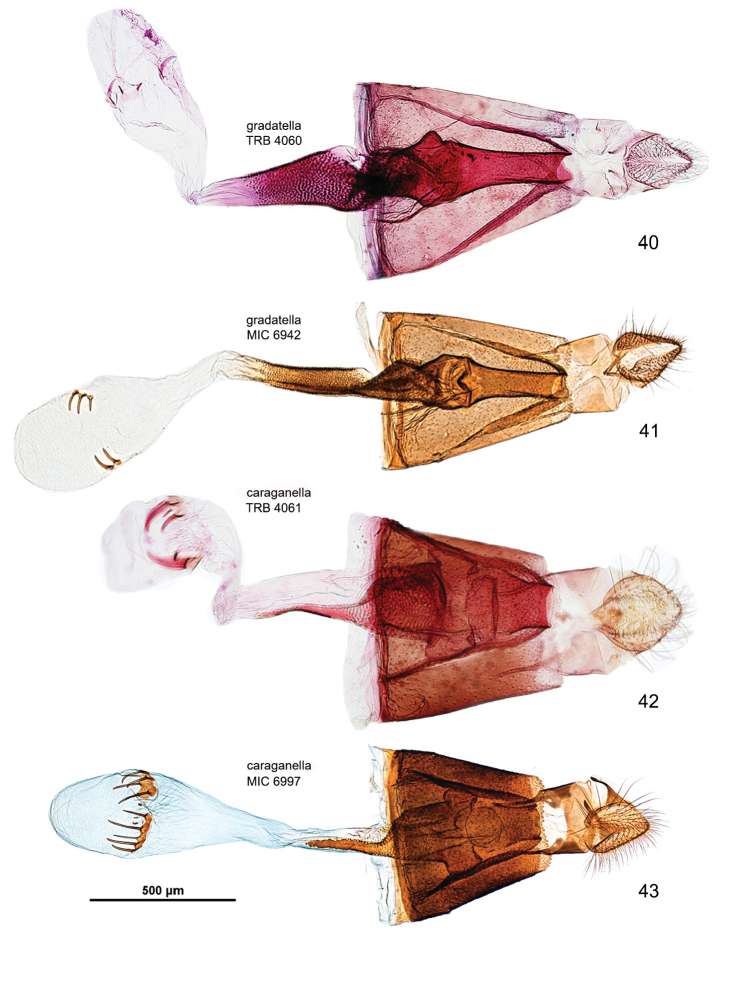
Female genitalia of *Micrurapteryx*. **40** *Micrurapteryx gradatella* (slide TRB4060) (Norway, Elverum) **41** *Micrurapteryx gradatella* (slide MIC6942, specimen CNCLEP00122240) (Norway, Norvegica) **42** *Micrurapteryx caraganella* (slide TRB4061, specimen NK415) (Russia, Krasnoyarsk) **43** *Micrurapteryx caraganella* (slide MIC6997, specimen CNCLEP00132306) (Russia, Omsk). Scale bars: 500 µm (**40, 41, 43**), 200 µm (**42**).

**Figures 44–48.**
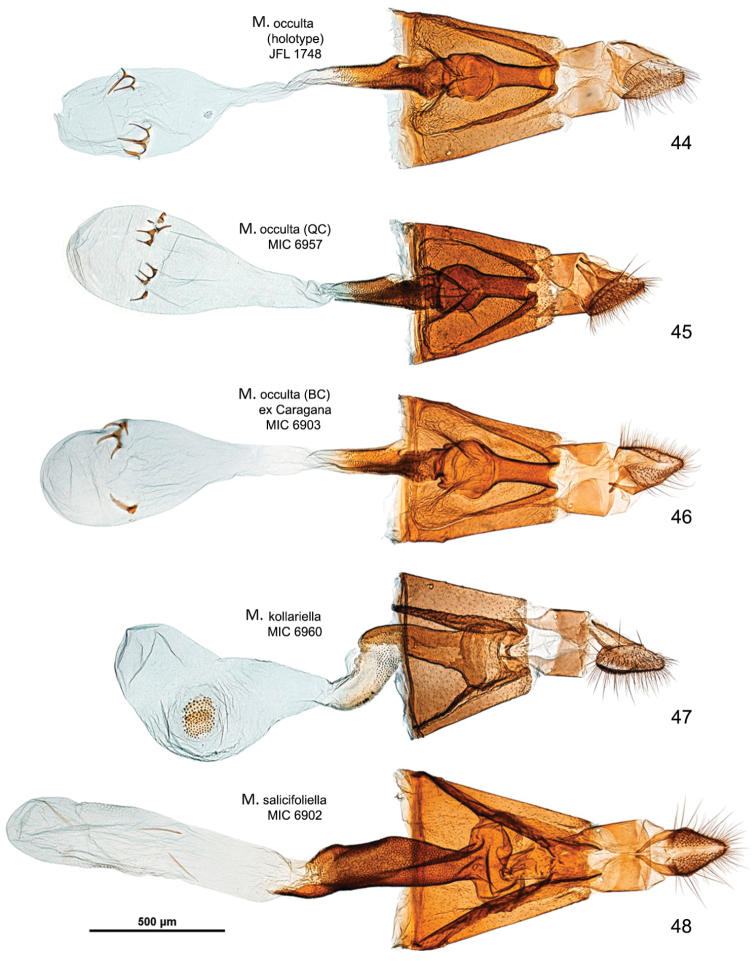
Female genitalia of *Micrurapteryx*. **44** *Micrurapteryx occulta* holotype (slide JFL1748, specimen CNCLEP00123636) (USA, Kentucky) **45** *Micrurapteryx occulta* (slide MIC6957, specimen CNCLEP00007544) (Canada, Quebec) **46** *Micrurapteryx occulta* (slide MIC6903, specimen CNCLEP00117698) (ex *Caragana*, Canada, British Columbia) **47** *Micrurapteryx kollariella* (slide MIC6960, specimen CNCLEP00123698) (Germany, Berlin) **48** *Micrurapteryx salicifoliella* (slide MIC6902, specimen CNCLEP00026530) (Canada, Yukon). Scale bars: 500 µm.

**Figures 49–54.**
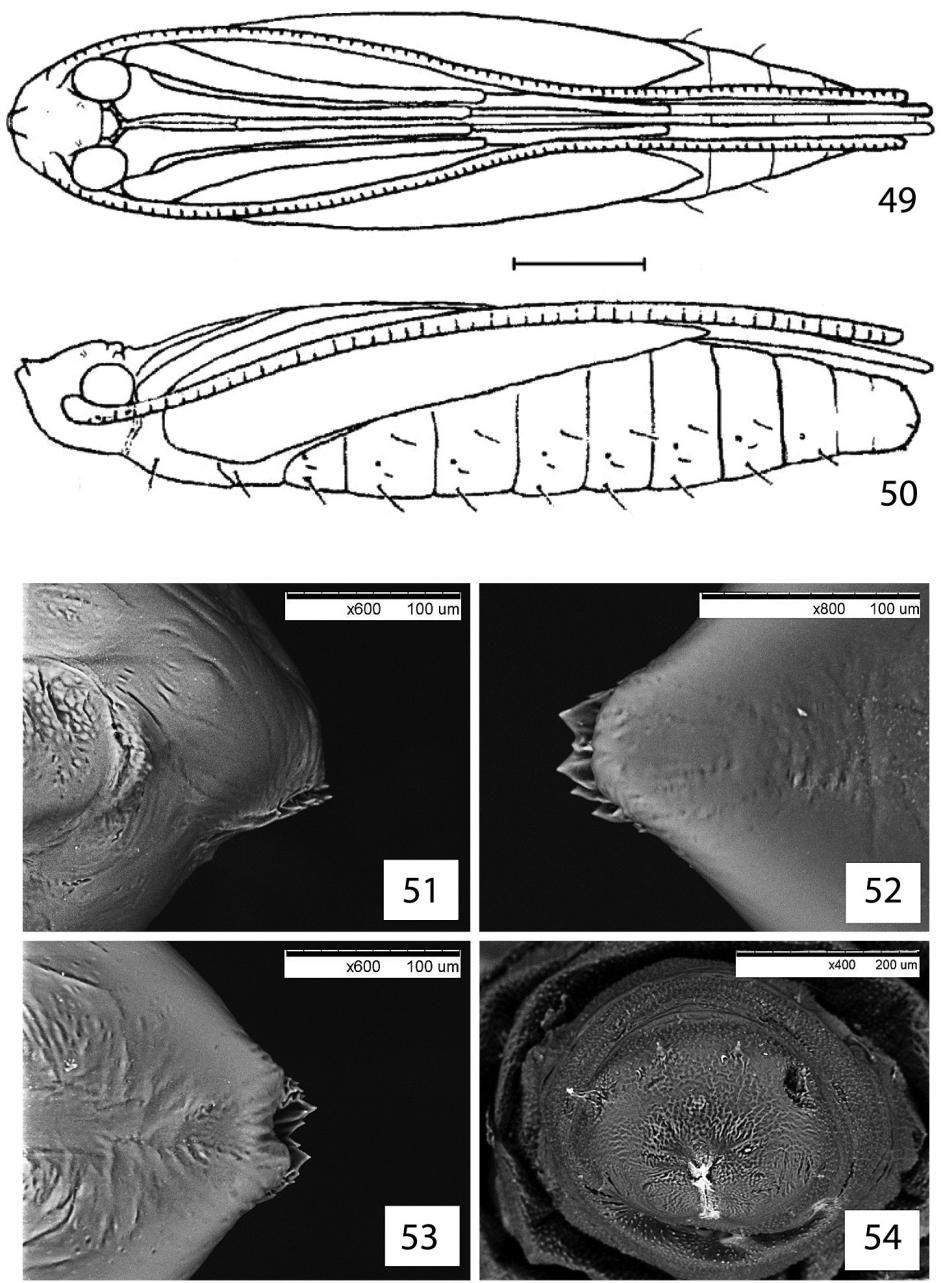
Pupa of *Micrurapteryx caraganella* sp. n. **49** ventral view **50** lateral view (scale 0.8 mm) **51** frontal process (cocoon cutter), lateral view **52** dorsal view of Fig. 51 **53** ventral view of Fig. 51 **54** cremaster spines of X abdominal segment.

**Figures 55–58.**
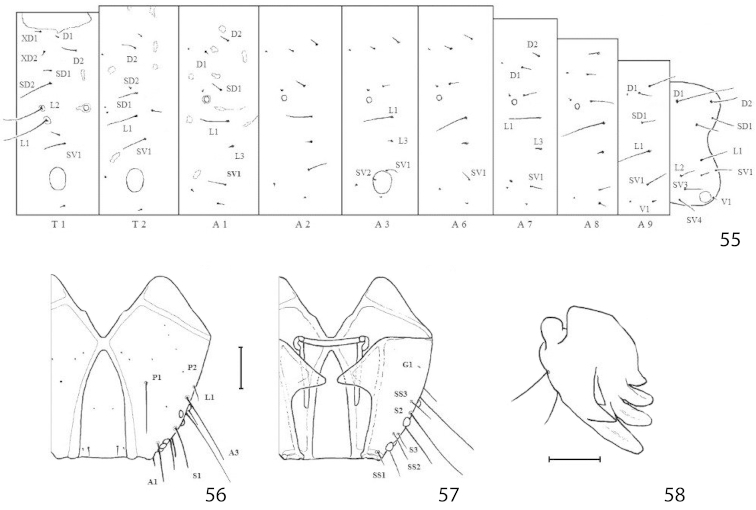
Chaetotaxy of last instars larva of *Micrurapteryx caraganella* sp. n. **55** lateral schematic of prothorax, mesothorax, and abdominal segments **56** dorsal view of head **57** ventral view of head (scale bar = 0.1 mm) **58** mandible (scale bar = 0.03 mm).

**Figures 59–64.**
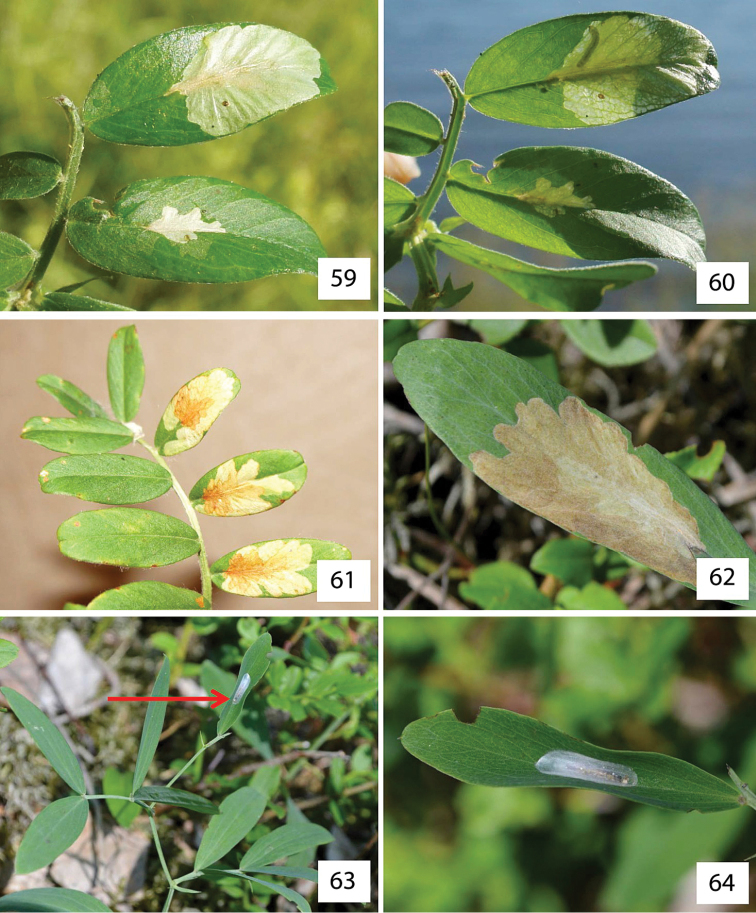
Life history of *Micrurapteryx gradatella* in Eurasia. **59–60** mines on *Vicia amoena* **61** abandoned mines on *Vicia amoena* **62** blotch mines on upperside of the leaves **63–64** pupation on the upperside of the leaf and the cocoon on *Lathyrus linifolius. Collection sites*: **59–60** Russia, Krasnoyarsk, Yenisei river bank, near village Borovoe, 5.VII.2015 **61** Russia, Krasnoyarsk, Yenisei river bank, near Karaulnaya, 26.VI.2015 **63–64** Finland, Turku, 18.VI.2014.

**Figures 65–76.**
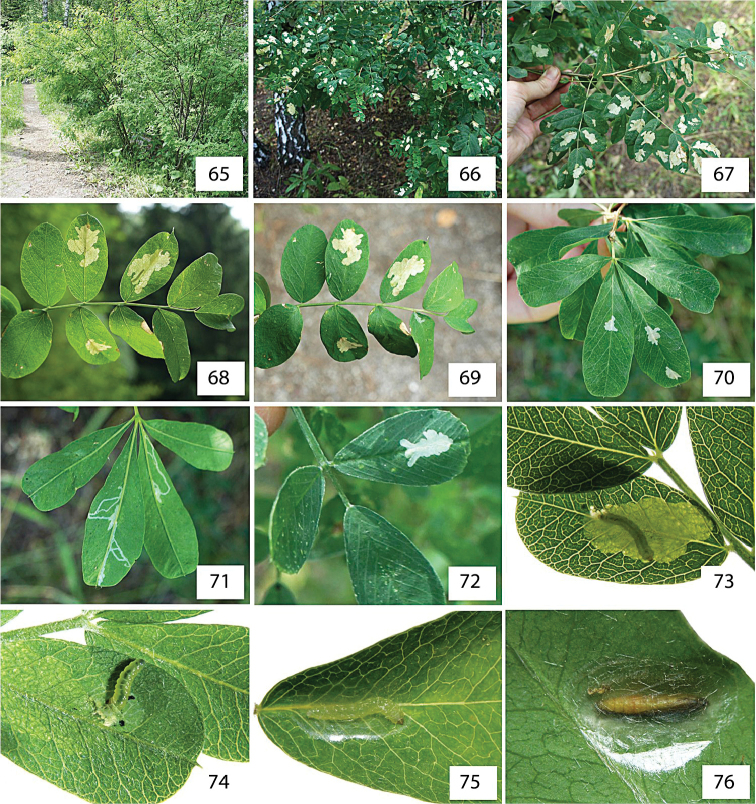
Life history of *Micrurapteryx caraganella* sp. n. in Siberia, Russia. **65** the species’ habitat **66–67** heavily defoliated bushes of *Caragana arborescens* **68–69** blotch mines on the upperside of the leaf, at transmitted light, with visible larva in one of the mines **70–71** mines on *Caragana frutex*, with long initial tunnels on the low side of the leaf (71) **72** mine on the leaf of *Medicago sativa* **73** larvae ejecting fecal pellets out of the leaf mine by protruding rear part of the body through a slit on low side of the leaf on *Caragana boisii* **74** larva vacating the mine on the low side of the leaf **75** larva spinning the cocoon on upper side of the leaf along the midrib **76** pupa in the transparent cocoon on lower side, perpendicular to the midrib. *Collection sites*: **65, 68, 69** Novosibirsk, Central Siberian botanical garden SB RAS, *Caragana arborescens*, 08.VIII.2012 **73, 74** same place, *Caragana boisii*, 14.VI.2012 **66, 67** Omsk, Victory Park, *Caragana arborescens*, 23.VII.2015 **70, 71** same place and date, *Caragana frutex*; **72** same place and date, *Medicago sativa* **75, 76** Krasnoyarsk, Akademgorodok, the left bank of the river Yenisei, *Caragana arborescens*, 15.VII.2013.

**Figures 77–78.**
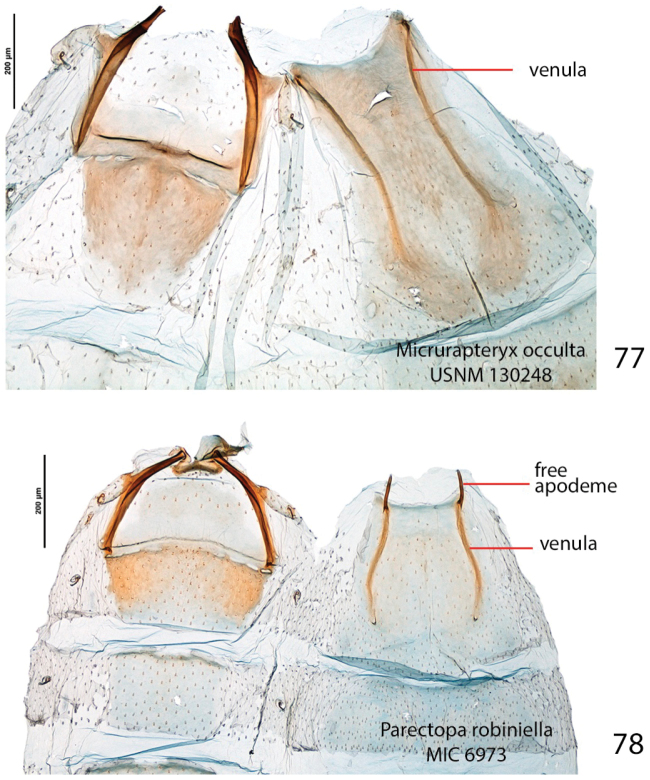
Comparison of male abdominal segments 1–2 of *Micrurapteryx* vs *Parectopa*. **77** *Micrurapteryx occulta* (slide USNM130248, specimen USNMENT00657165) (USA, California) **78** *Parectopa robiniella* (slide MIC6973, specimen CNCLEP00083022) (USA, Maryland). Scale bars: 200 µm.

**Figures 79–80.**
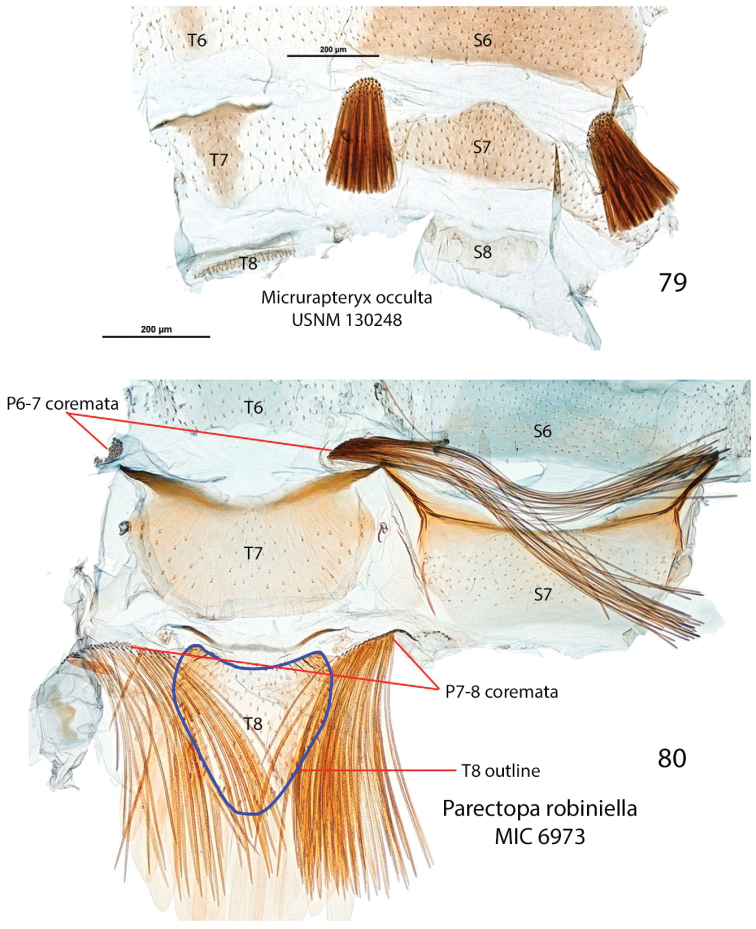
Comparison of male abdominal segments 6–8 of *Micrurapteryx* vs *Parectopa*. **79** *Micrurapteryx occulta* (slide USNM130248, specimen USNMENT00657165) (USA, California) **80** *Parectopa robiniella* (slide MIC6973, specimen CNCLEP00083022) (USA, Maryland). Scale bars: 200 µm.

**Figures 81–82.**
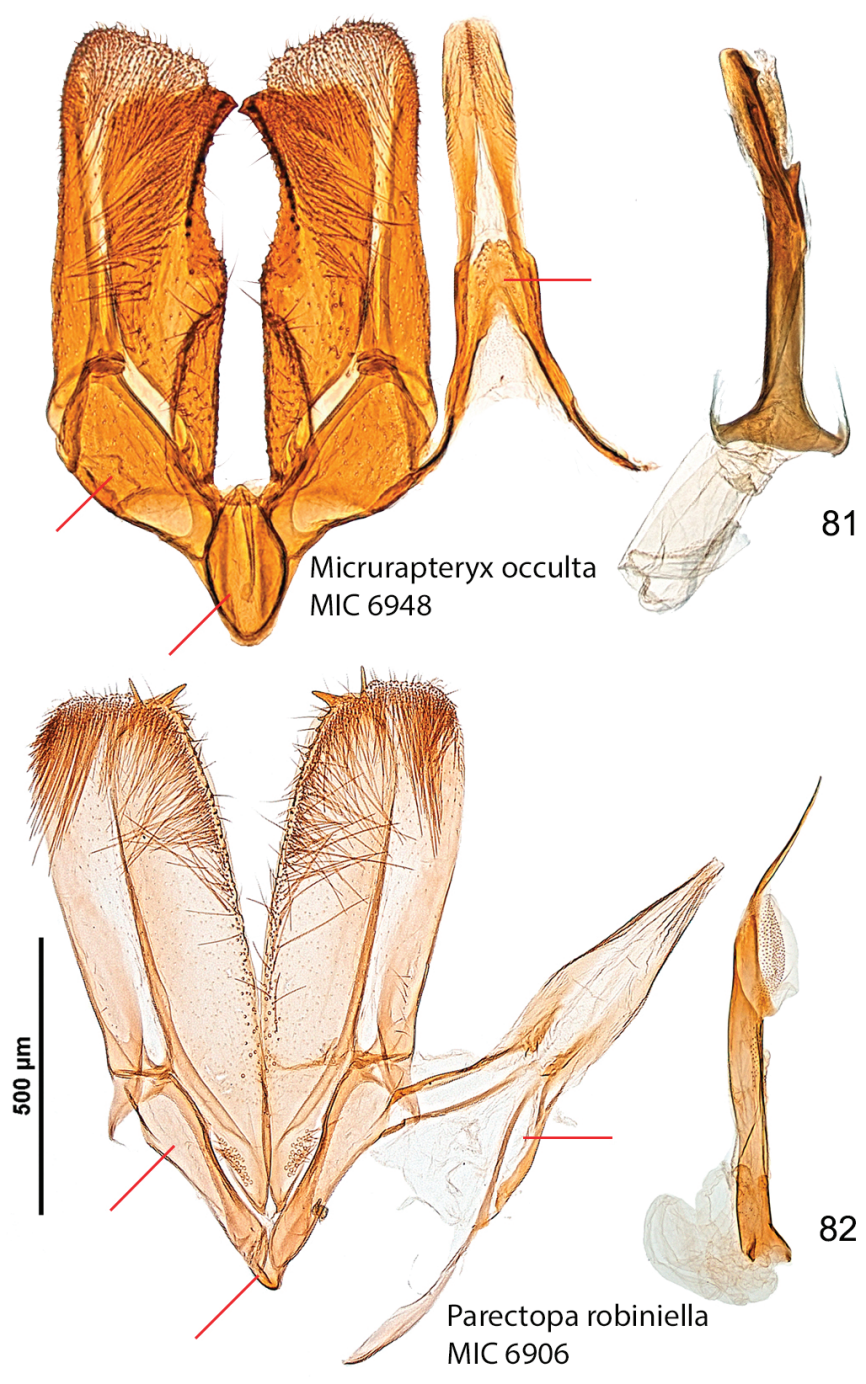
Comparison of male genitalia and phallus of *Micrurapteryx* vs *Parectopa*; red arrows point at distinctive features; phallus with dorsal side oriented to the right. **81** *Micrurapteryx occulta* (slide MIC6948, specimen AC006119) (Canada, Quebec) **82** *Parectopa robiniella* (slide MIC6906, specimen CNCLEP00083021) (USA, Maryland). Scale bars: 500 µm.

**Figures 83–86.**
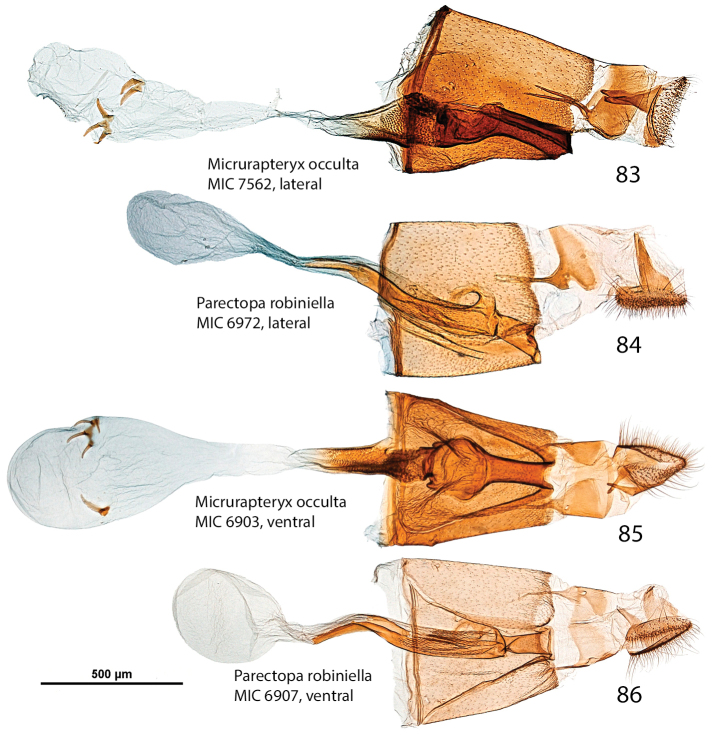
Comparison of female genitalia and phallus of *Micrurapteryx* vs *Parectopa*; lateral aspect with ventral side oriented downward. **83** *Micrurapteryx occulta*, lateral aspect (slide MIC7562, specimen BIOUG16843-E04) (Canada, Yukon, Ivvavik National Park) **84** *Parectopa robiniella*, lateral aspect (slide MIC6973, specimen CNCLEP00083022) (USA, Maryland) **85** *Micrurapteryx occulta*, ventral aspect (slide MIC6903, specimen CNCLEP00117698) (Canada, British Columbia) **86** *Parectopa robiniella*, ventral aspect (slide MIC6907, specimen CNCLEP00121057) (Canada, Nova Scotia, Smiths Cove). Scale bars: 500 µm.

## Supplementary Material

XML Treatment for Micrurapteryx gradatella

XML Treatment for Micrurapteryx caraganella

XML Treatment for Micrurapteryx occulta

## Supplementary materials

Supplementary material 1 Tables S1–S5 Data type: Tables Explanation note:
**Table S1.** Host plant range of *Micrurapteryx* species. The table provides data on host plants of all *Micrurapteryx* spp. of the Holarctic Region.
**Table S2.** Specimens of *Micrurapteryx* and *Parectopa* which were examined morphologically but not DNA barcoded. The table provides the list of specimens which were examined morphologically but not DNA barcoded. Where pertinent, genitalia slide numbers and sex are given in the table.
**Table S3.** Diagnostic substitutions in COI barcode sequences of *Micrurapteryx caraganella* and *Micrurapteryx gradatella*. The table provides diagnostic substitutions in COI barcode fragment allowing to distinguish *Micrurapteryx caraganella* from *Micrurapteryx gradatella*.
**Table S4.** Diagnostic substitutions in histone H3 and 28S sequences of *Micrurapteryx caraganella* and *Micrurapteryx gradatella*. The table provides diagnostic substitutions in histone H3 and 28S sequences allowing to distinguish *Micrurapteryx caraganella* from *Micrurapteryx gradatella*.
**Table S5.** Genital characters of *Micrurapteryx* and *Parectopa* extracted from Vári’s (1961) generic descriptions. The table compares male and female genital characters of *Micrurapteryx* and *Parectopa*. This dataset is made available under the Open Database License (http://opendatacommons.org/licenses/odbl/1.0/). The Open Database License (ODbL) is a license agreement intended to allow users to freely share, modify, and use this Dataset while maintaining this same freedom for others, provided that the original source and author(s) are credited.Natalia Kirichenko, Paolo Triberti, Marko Mutanen, Emmanuelle Magnoux, Jean-François Landry, Carlos Lopez-Vaamonde

Supplementary material 2 Figs S01–S12 Data type: JPG image file Explanation note: *Micrurapteryx occulta*, male genitalia. The plate shows intraspecific variation of male genitalia in *Micrurapteryx occulta*. This dataset is made available under the Open Database License (http://opendatacommons.org/licenses/odbl/1.0/). The Open Database License (ODbL) is a license agreement intended to allow users to freely share, modify, and use this Dataset while maintaining this same freedom for others, provided that the original source and author(s) are credited.Natalia Kirichenko, Paolo Triberti, Marko Mutanen, Emmanuelle Magnoux, Jean-François Landry, Carlos Lopez-Vaamonde

Supplementary material 3 Figs S13–S24 Data type: JPG image file Explanation note: *Micrurapteryx occulta*, male genitalia. The plate shows intraspecific variation of male genitalia in *Micrurapteryx occulta*. This dataset is made available under the Open Database License (http://opendatacommons.org/licenses/odbl/1.0/). The Open Database License (ODbL) is a license agreement intended to allow users to freely share, modify, and use this Dataset while maintaining this same freedom for others, provided that the original source and author(s) are credited.Natalia Kirichenko, Paolo Triberti, Marko Mutanen, Emmanuelle Magnoux, Jean-François Landry, Carlos Lopez-Vaamonde

Supplementary material 4 Figs S25–S36 Data type: JPG image file Explanation note: *Micrurapteryx occulta* and *Micrurapteryx caraganella*, male genitalia. The plate shows intraspecific variation of male genitalia in *Micrurapteryx occulta*. This dataset is made available under the Open Database License (http://opendatacommons.org/licenses/odbl/1.0/). The Open Database License (ODbL) is a license agreement intended to allow users to freely share, modify, and use this Dataset while maintaining this same freedom for others, provided that the original source and author(s) are credited.Natalia Kirichenko, Paolo Triberti, Marko Mutanen, Emmanuelle Magnoux, Jean-François Landry, Carlos Lopez-Vaamonde

Supplementary material 5 Figs S37–S44 Data type: JPG image file Explanation note: *Micrurapteryx occulta*, female genitalia. The plate shows intraspecific variation of female genitalia in *Micrurapteryx occulta*. This dataset is made available under the Open Database License (http://opendatacommons.org/licenses/odbl/1.0/). The Open Database License (ODbL) is a license agreement intended to allow users to freely share, modify, and use this Dataset while maintaining this same freedom for others, provided that the original source and author(s) are credited.Natalia Kirichenko, Paolo Triberti, Marko Mutanen, Emmanuelle Magnoux, Jean-François Landry, Carlos Lopez-Vaamonde

Supplementary material 6 Figs S45–S52 Data type: JPG image file Explanation note: *Micrurapteryx occulta*, female genitalia. The plate shows intraspecific variation of female genitalia in *Micrurapteryx occulta*. This dataset is made available under the Open Database License (http://opendatacommons.org/licenses/odbl/1.0/). The Open Database License (ODbL) is a license agreement intended to allow users to freely share, modify, and use this Dataset while maintaining this same freedom for others, provided that the original source and author(s) are credited.Natalia Kirichenko, Paolo Triberti, Marko Mutanen, Emmanuelle Magnoux, Jean-François Landry, Carlos Lopez-Vaamonde
