# Supplementary material for: Systematics and biology of some species of Micrurapteryx Spuler (Lepidoptera, Gracillariidae) from the Holarctic Region, with re-description of M. caraganella (Hering) from Siberia
Source: Zookeys. 2016 Apr 11;(579):99–156. doi: 10.3897/zookeys.579.7166 (PMC4829971; doi:10.3897/zookeys.579.7166)
Supplement: Supplementary material 1 — Tables S1–S5 [file zookeys-579-099-s001.pdf]

Supplementary materials

ZooKeys

Systematics and biology of some species of *Micrurapteryx* Spuler (Lepidoptera, Gracillariidae) from the Holarctic Region, with re-description of *M. caraganella* (Hering) from Siberia

Natalia Kirichenko[1-3], Paolo Triberti[4], Marko Mutanen[5], Emmanuelle Magnoux[3], Jean-François Landry[6], Carlos Lopez-Vaamonde[3, 7]

[1]Sukachev Institute of Forest SB RAS, Akademgorodok 50/28, 660036, Krasnoyarsk, Russia

[2]Siberian Federal University, 79 Svobodny pr., 660041, Krasnoyarsk, Russia

[3]INRA, UR0633 Zoologie Forestière, F-45075 Orléans, France

[4]Museo Civico di Storia Naturale, Lungadige Porta Vittoria 9, I37129, Verona, Italy

[5]Department of Genetics and Physiology, P.O. Box 3000, FI-90014 University of Oulu, Finland

[6]Agriculture and Agri-Food Canada, Ottawa Research and Development Centre, Central Experimental Farm, Ottawa, Ontario K1A 0C6, Canada

[7]Institut de Recherche sur la Biologie de l’Insecte, CNRS UMR 7261, Université François-Rabelais de Tours, UFR Sciences et Techniques, 37200 Tours, France

Corresponding author: Natalia Kirichenko (nkirichenko@yahoo.com)

Table S1. Host plant range of *Micrurapteryx* species.

№	***Micrurapteryx* species**[1]	**Host plant family**	**Host plant species**[2]	**Reference**[3]
1	*M. bidentata* Noreika, 1992	not known	—	Noreika and Puplesis (1992)
2	*M. fumosella* Kuznetzov & Tristan, 1985	Fabaceae	*Astragalus alpinus* L., *Astragalus* sp., *Melilotus albus* Medik., *Melilotus* sp., *Trifolium pratense* L., *Trifolium* sp., *Vicia cracca* L., *Vicia* sp.	Kuznetzov and Tristan (1985)
3	*M. gerasimovi* Ermolaev, 1982	Fabaceae	*Melilotus suaveolens* Ledeb., *Vicia cracca* L.	Ermolaev (1982)
4	*M. gradatella* (Herrich-Schäffer, 1855)	Fabaceae	*Lathyrus linifolius* (Reichard) Bässler [Syn. *Lathyrus montanus* Bernh., *L. linifolius* subsp. *montanus* (Bernhardi) Bässler, *Orobus tuberosus* L.], *L. tuberosus* L. and *Vicia sepium* L.	Hering (1957); Noreika (1997); De Prins and De Prins (2015)
			Vicia amoena	present paper
5	*M. kollariella* (Zeller, 1839)	Fabaceae	*Chamaecytisus ruthenicus* (Fisch. ex Woł.) Klásk. [Syn. *Cytisus ruthenicus* Fisch. ex Woł.], *Cytisophyllum sessilifolium* (L.) O. Lang [Syn. *Cytisophyllum sessilifolius* (L.) O. Lang], *Cytisus hirsutus* L. [Syn. *Chamaecytisus hirsutus* (L.) Link; *Cytisus capitatus* Scop.], *C. ratisbonensis* Schaeff. [Syn. *Chamaecytisus ratisbonensis* (Schaeff.)Rothm.], *C. scoparius* (L.) Link, *Cytisus* sp., *Genista pilosa* L., *G. tinctoria* L., *Genista* sp., *Hippocrepis emerus* (L.) Lassen [Syn. *Coronilla emerus* L.], *Laburnocytisus* sp., *Laburnum anagyroides* Medik., 1787, *Laburnum* sp., *Lembotropis nigricans* (L.) Griseb., *Lupinus* sp., *Petteria* sp., *Sarothamnus* sp.	Review in De Prins and De Prins (2015)
			Erroneously: *Astragalus, Glycyrrhiza, Styphnolobium, Thermopsis*[#]	Dovnar-Zapol'sliy (1969); Barakanova (1986)
6	*M. parvula* Amsel, 1935	not known	—	Kuznetzov and Tristan (1985)
7	*M. salicifoliella* (Chambers, 1872)	Salicaceae	*Salix spp.*(more than 15 species)	Review in De Prins and De Prins (2015)
8	*M. sophorella* Kuznetzov, 1979	not known	—; erroneously *Sophora* sp.[†]	Kuznetzov and Tristan (1985)

№	*Micrurapteryx* species[1]	Host plant family	Host plant species[2]	Reference[3]
9	*M. sophorivora* Kuznetzov & Tristan, 1985	Fabaceae	*Sophora* sp., *Robinia pseudoacacia* L.	Kuznetzov and Tristan (1985); Gencer and Seven (2005)
10	*M. tibetiensis* Bai & Li, 2013	not known	—	Bai (2013)
11	*M. tortuosella* Kuznetzov & Tristan, 1985	Fabaceae	*Lathyrus odoratus* L., *Lathyrus* sp., *Medicago sativa* L., *Medicago* sp., *Melilotus* sp.	Kuznetzov and Tristan (1985)
Additional species cited in our study				
12	*M. caraganella* (Hering, 1957)	Fabaceae	*Caragana arborescens* Lam., *C. frutex* (L.) K. Koch, *C. boisii* C. K. Schneid.), *Medicago sativa* L.	present paper
13	*M. occulta* (Braun, 1922)	Fabaceae	*Lathyrus japonicus* Willd. [Syn. *Lathyrus maritimus* (L.) Fr.], *Lathyrus* sp., *Melilotus albus* Medik., *Vicia caroliniana* Walter (type of *M. occulta*), "vetch" (type of *M. albicostella*), *Lupinus* sp., *Caragana* sp.	present paper

[1] Species covered by our study are underlined.

[2] In [], the earlier names of host plants are listed as they were cited in the original sources for *Micrurapteryx* spp. Actual names of the species are provided according to APG III system (theplantlist.org).

[#] Dovnar-Zapol'skiy (1969) recorded *Astragalus*, *Glycyrrhiza*, *Styphnolobium* and *Thermopsis* as host plants of *M. kollariella* based on leaf mines. However mine morphology does not seem to be species-specific in *Micrurapteryx* and Kuznetsov and Tristan (1985) doubted of the identification of *M. kollariella* based only on leaf mines and suggest that those plants are more likely the hosts of other *Micrurapteryx* species such as *M. fumosella*, *M. sophorella* or *M. sophorivora*. Therefore more work is needed to confirm the above mentioned plants as hosts of *M. kollariella*.

[†] Kuznetsov and Tristan (1985) indicated that *Sophora* sp. was erroneously attributed to *M. sophorella* in Kuznetsov (1979). *Sophora* sp. is a host of *M. sophorivora.*

— no data.

[3] Reference:

Bai HY (2013) A new species of *Micrurapteryx* Spuler (Lepidoptera: Gracillariidae) from Tibet, China. Entomological News 122(4): 324–327.

Barakanova (1986) Review of Lepidoptera found on Legumes in eastern Kirgizia. Entomologicheskoe Obozrenie 3: 503–512. [in Russian].

De Prins J, De Prins W (2015) Global Taxonomic Database of Gracillariidae (Lepidoptera) http://www.gracillariidae.net

Dovnar-Zapol'skiy DP (1969) Mining insects on plants of Kirgizia and adjacent territory. Ilim, Frunze, 148 pp. [in Russian].

Ermolaev VP (1982) A review of Gracillariidae (Lepidoptera) injurious to the legumenes in the south of the Primorye Territory, with description of two new species. Entomologicheskoe Obozrenie 61(3): 572–581. [in Russian].

Gencer L, Seven S (2005) Chalcidoid parasitoids of *Micrurapteryx sophorivora* (Lepidoptera: Gracillariidae) in Kuluncak, Turkey. Phytoprotection 86(2): 133–134.

Hering EM (1957) Bestimmungstabellen der Blattminen vom Europa einschliesslich des Mittelmeerbecken und der Kanarischen Inseln. 3 Bände. W. Junk, Gravenhage, 1185+221 pp.

Kuznetzov VI (1979) A review of the genera of Gracillariidae (Lepidoptera) of the Palaearctic fauna. Entomologicheskoe Obozrenie 58(4): 835–856. [in Russian].

Kuznetzov VI, Tristan NI (1985) A review of the leaf blotch miners of the genus *Micrurapteryx* Spuler (Lepidoptera Gracillariidae) of the Palaearctic fauna. Entomologicheskoe Obozrenie 64(1): 177–192. [in Russian].

Noreika R (1997) Gracillariidae. In: Ler PA (Ed) Key to the insects of the Russian Far East. Volume V. Trichoptera and Lepidoptera. Part 1: 373–429. [in Russian].

Noreika R, Puplesis R (1992) Description of new species of moths of the family Gracillariidae (Lepidoptera) from Azerbaijan and Middle Asia and synonymy of *Gracillaria impictipennella* Grsm. Entomologicheskoe Obozrenie 71(2):414–421. [in Russian].

Table S2. Specimens of *Micrurapteryx* and *Parectopa,* which were examined morphologically but not DNA barcoded.

№	Species, stage (P-pupa, L – larva, A – adult), number of studied specimens in (), specimen ID and genitalia preparation in []	Host plant	Collector	Collection[1]	Collection date	Country	Locality	Latitude	Longitude	Elevation (m)
	Micrurapteryx caraganella									
1	P (1)	*Caragana arborescens*	Kirichenko N.	MSNV	03.VIII.2011	Russia	Novosibirsk, CSBG SB RAS	55.82	83.10	155
2	L (1)	*C. boisii*	Kirichenko N.	MSNV	06.VI.2012	Russia	Novosibirsk, CSBG SB RAS	55.82	83.10	155
3	L (6)	*C. arborescens*	Kirichenko N.	MSNV	12.VII.2013	Russia	Krasnoyarsk, Akademgorodok	55.99	92.77	256
4	A (1), Kr-19-13-1 holotype	*C. arborescens*	Kirichenko N.	MSNV	12.VII.2013	Russia	Krasnoyarsk, Akademgorodok	55.99	92.77	256
5	A (1), [MIC6941♂]	*C. arborescens*	Kirichenko N.	MSNV	12.VII.2013	Russia	Krasnoyarsk, Akademgorodok	55.99	92.77	256
6	A (1), CNCLEP00122241, [MIC6940♂]	*C. arborescens*	Kirichenko N.	MSNV	18.VIII.2013	Russia	Krasnoyarsk, Akademgorodok	55.99	92.77	256
7	P (6)	*C. arborescens*	Kirichenko N.	MSNV	11.VII.2013	Russia	Krasnoyarsk, Akademgorodok	55.99	92.77	256
8	A (1), [NK-186-15-1♂]	*C. abrorescens*	Kirichenko N.	SIF	23.VII.2015	Russia	Omsk, Victory park	54.99	73.34	69
9	A (1), [NK-186-15-2♂]	*C. abrorescens*	Kirichenko N.	SIF	23.VII.2015	Russia	Omsk, Victory park	54.99	73.34	69
10	L (1), NK-186-15	*C. arborescens*	Kirichenko N.	SIF	23.VII.2015	Russia	Omsk, Victory park	54.99	73.34	69
11	A (2), CNCLEP00132306–307, [MIC6997♀]	*C. arborescens*	Kirichenko N.	CNC	31.VII.2015	Russia	Omsk, Victory park	54.99	73.34	69
12	L (1), NK-209-15	*C. arborescens*	Kirichenko N.	SIF	24.VII.2015	Russia	Tyumen: Zatyumenskiy park	57.16	64.47	80
13	L (1), NK-212-15	*C. arborescens*	Kirichenko N.	SIF	25.VII.2015	Russia	Tobolsk: Ermak park	58.18	68.23	41
14	L (1), NK-223-15	*C. arborescens*	Kirichenko N.	SIF	27.VII.2015	Russia	Barnaul: Izymrudniy park	53.36	83.78	192
15	L (1), NK-184-15-1	*C. frutex*	Kirichenko N.	SIF	23.VII.2015	Russia	Omsk, Victory park	54.99	73.34	69

№	Species, stage (P-pupa, L – larva, A – adult), number of studied specimens in (), specimen ID and genitalia preparation in []	Host plant	Collector	Collection[1]	Collection date	Country	Locality	Latitude	Longitude	Elevation (m)
16	A (1), [NK-184-15♀]	*C. frutex*	Kirichenko N.	SIF	23.VII.2015	Russia	Omsk, Victory park	54.99	73.34	69
17	A (1), [NK-184-15-1♂]	*C. frutex*	Kirichenko N.	SIF	23.VII.2015	Russia	Omsk, Victory park	54.99	73.34	69
18	A (1), [NK-184-15-2♂]	*C. frutex*	Kirichenko N.	SIF	23.VII.2015	Russia	Omsk, Victory park	54.99	73.34	69
19	A (1), [TRB3995♂]	*C. arborescens*	Kirichenko N.	MSNV	12.VII.2013	Russia	Krasnoyarsk, Akademgorodok	55.99	92.77	256
	Micrurapteryx gradatella									
20	A (1), [TRB755♂]	—	Predota	MSNV	1911	Romania	Mezöseg	44.28	24.62	401
21	A (2), [MIC6942 ♂]	*Lathyrus montanus*	Larsen K.	MSNV (1) CNC(1)	20.VI.1961	Norway	HEs, Norvegica	60.92	11.48	337
22	A (1), [TRB4060 ♀]	*L. montanus*	Karsholt O.	MSNV	28.VI.1981	Norway	HEs, Elverum, Hernes	60.92	11.48	337
23	P (5)	*L. linifolius*	Itamies J.	MSNV	12.VI.2000	Finland	Turku	60.43	22.25	25
24	A (1), [TRB4081♂]	*L. linifolius*	Mutanen M.	MSNV	VI.2000	Finland	Turku	60.43	22.25	25
25	A (1), [TRB4091♂]	*L. linifolius*	Mutanen M.	MSNV	VI.2000	Finland	Turku	60.43	22.25	25
26	A (1), [TRB4095♂]	*L. linifolius*	Mutanen M.	MSNV	VI.2000	Finland	Turku	60.43	22.25	25
27	A (1), [NK-82-15-1♂]	*Vicia amoena*	Kirichenko N.	SIF	3.VII.2015	Russia	Krasnoyarsk, Yenisei; near Karaulnaya	55.98	92.75	208
28	A (1), [NK-82-15-2♂]	*V. amoena*	Kirichenko N.	SIF	3.VII.2015	Russia	Krasnoyarsk, Yenisei; near Karaulnaya	55.98	92.75	208
	Micrurapteryx kollariella									
29	A (1), CNCLEP00123697, [MIC 6959 ♂]	*Cytisus sp.*	Hering M.	CNC	—	Germany	Berlin	52.51	13.40	48
30	A (1), CNCLEP00123698, [MIC 6960 ♀]	*Cytisus sp.*	Hering M.	CNC	—	Germany	Berlin	52.51	13.40	48
	Micrurapteryx occulta									
31	A (1), CNCLEP00123986	—	Braun A.F.	CNC	29.IV.1905	USA	Ohio, Cincinnati	39.10	-84.50	148
32	A (1), CNCLEP00123636	*Vicia caroliniana*	Braun A.F.	ANSP	1921	USA	Kentucky	37.78	-84.34	293

№	Species, stage (P-pupa, L – larva, A – adult), number of studied specimens in (), specimen ID and genitalia preparation in []	Host plant	Collector	Collection[1]	Collection date	Country	Locality	Latitude	Longitude	Elevation (m)
	Holotype, [JFL 1748 ♀]									
33	A (1), CNCLEP00123635 Holotype (*Parectopa albicostella*), [DRD 3764 ♂]	"vetch"	Braun A.F	ANSP	1924	USA	Utah	40.65	-111.85	1456
34	A (1), CNCLEP00123982	–	Petch C.E.	CNC	14.VIII.1924	Canada	Québec, Hemmingford	45.03	-74.57	44
35	A (1), CNCLEP00123990	*Melilotus alba*	White R.M.	CNC	24.IX.1925	Canada	Manitoba, Aweme	49.71	-99.60	365
36	A (1), CNCLEP00123989	*M. alba*	White R.M.	CNC	9.IX.1925	Canada	Manitoba, Aweme	49.71	-99.60	365
37	A (1), CNCLEP00123992	*M. alba*	White R.M.	CNC	2.VII.1926	Canada	Manitoba, Aweme	49.71	-99.60	365
38	A (1), CNCLEP00123991	*M. alba*	White R.M.	CNC	2.VII.1926	Canada	Manitoba, Aweme	49.71	-99.60	365
39	A (1), CNCLEP00123994, [MIC 6963 ♀]	*M. alba*	White R.M.	CNC	3.VII.1926	Canada	Manitoba, Aweme	49.71	-99.60	365
40	A (1), CNCLEP00123993	*M. alba*	White R.M.	CNC	3.VII.1926	Canada	Manitoba, Aweme	49.71	-99.60	365
41	A (1), CNCLEP00123995	*M. alba*	White R.M.	CNC	4.VII.1926	Canada	Manitoba, Aweme	49.71	-99.60	365
42	A (1), CNCLEP00123997, [MIC 6962 ♂]	*M. alba*	White R.M.	CNC	5.VII.1926	Canada	Manitoba, Aweme	49.71	-99.60	365
43	A (1), CNCLEP00123998	*M. alba*	White R.M.	CNC	6.VII.1926	Canada	Manitoba, Aweme	49.71	-99.60	365
44	A (1), CNCLEP00123999	*M. alba*	White R.M.	CNC	7.VII.1926	Canada	Manitoba, Aweme	49.71	-99.60	365
45	A (1), CNCLEP00123987	—	McDunnough J.	CNC	9.VI.1926	Canada	British Columbia, Lilloeet, Seton Lake	50.67	-122.10	500
46	A (1), CNCLEP00124000, [MIC 6978 ♂]	*M. alba*	White R.M.	CNC	9.VII.1926	Canada	Manitoba, Aweme	49.71	-99.60	365
47	A (1), CNCLEP00123983	—	Young C.H.	CNC	22.VIII.1932	Canada	Ontario, Ottawa	45.20	-75.70	89
48	A (1), CNCLEP00123984	—	Walley G.S.	CNC	30.IV.1933	Canada	Ontario, Constance Bay	45.48	-76.04	66
49	A (1),CNCLEP00123988	—	Jacob J.K.	CNC	1.IX.1938	Canada	British Columbia, Jesmond	51.24	-121.95	1218
50	A (1), CNCLEP00123985	—	Beebe R.	CNC	6.VIII.1955	USA	Kentucky , Rockcastle Co.	37.30	-84.30	339
51	A (1), CNCLEP00123996, [MIC 2151 ♂]	*M. alba*	White R.M.	CNC	3.VII.1926	Canada	Manitoba, Aweme	49.71	-99.60	365

№	Species, stage (P-pupa, L – larva, A – adult), number of studied specimens in (), specimen ID and genitalia preparation in []	Host plant	Collector	Collec-tion[1]	Collection date	Country	Locality	Lati-tude	Longi-tude	Eleva-tion (m)
52	A (1), CNCLEP00123692	—	Freeman & Lewis	CNC	26.VII.1956	Canada	Ontario, Normandale	42.71	-80.31	188
53	A (1), CNCLEP00117700, [MIC 6966 ♀]	*Caragana arborescens*	Freeman & Lewis	CNC	1957	Canada	British Columbia, Lumby	50.25	-118.97	500
54	A (1), CNCLEP00117699	*C. arborescens*	Freeman & Lewis	CNC	1957	Canada	British Columbia, Lumby	50.25	-118.97	500
55	A (1), CNCLEP00117698, [MIC 6903 ♀]	*C. arborescens*	Freeman & Lewis	CNC	1957	Canada	British Columbia, Lumby	50.25	-118.97	500
56	A(1), CNCLEP00123694, [MIC 6958 ♀]	*Melilotus alba*	Freeman & Lewis	CNC	3.VII.1957	Canada	British Columbia, Vernon	50.26	-119.27	390
57	A (1), CNCLEP00123694, [MIC 6958 ♀]	*M. alba*	Freeman	CNC	3.VII.1957	Canada	British Columbia, Vernon	50.28	-119.27	390
58	A (1), CNCLEP00123695	*M. alba*	Freeman & Lewis	CNC	12.VII.1957	Canada	British Columbia, Coldstream	50.22	-119.23	390
59	A (1), CNCLEP00123693	*M. alba*	Freeman & Lewis	CNC	15.VII.1957	Canada	British Columbia, Vernon	50.26	-119.27	390
60	A (1), CNCLEP00123687	—	Landry J.-F.	CNC	9.IV.1987	Canada	Ontario, Ottawa	45.44	-75.61	82
61	A (1), CNCLEP00123688	—	Kritsch D.	CNC	2.XII.1987	Canada	Ontario, Ottawa	45.39	-75.71	85
62	A (1), CNCLEP00123686	—	Dang P.T.	CNC	20.IV.1987	Canada	Ontario, Ottawa	45.31	-75.72	91
63	A (1), CNCLEP00123685	—	Dang P.T.	CNC	20.IV.1987	Canada	Ontario, Ottawa	45.31	-75.72	91
64	A (1), CNCLEP00123681	—	Landry J.-F.	CNC	27.VII.1990	Canada	Quebec, Gatineau, Aylmer	45.40	-75.85	84
65	A (1), CNCLEP00123684, [MIC 6951 ♂]	—	Landry J.-F.	CNC	13.IX.1991	Canada	Quebec, Eardley	45.57	-76.10	100
66	A (1), CNCLEP00123676	—	Landry B.	CNC	6.V.1993	Canada	Quebec, Gatineau, Aylmer	45.44	-75.85	133
67	A (1)*, USNMENT00657161, [USNM 130245 ♀]	*Lathyrus sp.*	Davis D.R.	USNM	14.IX.1993	USA	California	39.92	-120.06	1857
68	A (1), CNCLEP00123677, [MIC 6950 ♂]	—	Landry B.	CNC	12.VII.1995	Canada	Quebec, Gatineau, Aylmer	45.40	-75.85	80

№	Species, stage (P-pupa, L – larva, A – adult), number of studied specimens in (), specimen ID and genitalia preparation in []	Host plant	Collector	Collection[1]	Collection date	Country	Locality	Latitude	Longitude	Elevation (m)
69	A (1), CNCLEP00123680	—	Landry B.	CNC	11.VI.1997	Canada	Quebec, Gatineau, Aylmer	45.40	-75.84	90
70	A (1), CNCLEP00123679	—	Landry B.	CNC	1.IV.1998	Canada	Quebec, Gatineau, Aylmer	45.40	-75.84	90
71	A (1), CNCLEP00123678	—	Landry B.	CNC	13.VII.1998	Canada	Quebec Abitibi-Ouest, Lasarre	48.80	-79.20	276
72	A (1), CNCLEP00123682	*Melilotus sp*	Landry J.-F.	CNC	27.VII.1998	Canada	Quebec, Gatineau, Aylmer	45.40	-75.85	84
73	A (1), CNCLEP00123683	—	Landry J.-F.	CNC	14.VII.1999	Canada	Quebec, Pontiac, Breckenridge	45.47	-75.95	61
74	A (1)*, CNCLEP00007544, [MIC 6957 ♀]	—	Landry J.-F.	CNC	07.VIII.2001	Canada	Quebec, Gatineau, Aylmer	45.40	-75.85	80
75	A (1), CNCLEP00016559, [MIC 6901 ♀]	—	Landry J.-F.	CNC	9.V.2005	Canada	Quebec, Gatineau Park, Luskville Falls	45.53	-75.99	80
76	A (1)*, 113670446Jun2008	—	Lagasa E.H.	WSDA	06.VI.2008	USA	Washington	41.56	-119.75	1695
	Micrurapteryx salicifoliella									
77	A (1), CNCLEP00117661	*Salix sp.*	—	CNC	31.VII.1978	Canada	Ontario, Jellicoe	49.68	-87.53	330
78	A (1), CNCLEP00132256, [MIC 6976 ♂]	*Salix sp.*	Langor D.	CNC	8.VII.1994	Canada	Alberta, 30 km S High Level on Rte 35	57.92	-117.54	375
79	A (1), CNCLEP00123690, [MIC 6952 ♂]	—	Morton, J.K.	CNC	22.V.2000	Canada	Ontario, Manitoulin Island	45.68	-82.05	241
	Parectopa lespedezaefoliella									
80	A (1), CNCLEP00104018, [MIC 6975 ♀]	—	Braun A.F.	CNC	8.IX.1903	USA	Ohio, Cincinnati	39.10	-84.47	192
81	A (1), CNCLEP00104022, [MIC 6974 ♂]	*Desmodium paniculatum*	Freeman & Lewis	CNC	11.III.1958	Canada	Ontario, Normandale	42.71	-80.31	191

№	Species, stage (P-pupa, L – larva, A – adult), number of studied specimens in (), specimen ID and genitalia preparation in []	Host plant	Collector	Collec-tion[1]	Collection date	Country	Locality	Lati-tude	Longi-tude	Eleva-tion (m)
	Parectopa ononidis									
82	A (1), [NK-97-15-1♂]	*Trifolium pratense*	Kirichenko N.	SIF	5.VII.2015	Russia	Krasnoyarsk, Yenisei, Skala Berkut,	55.97	92.65	173
83	A (1), [NK-97-15-2♂]	*T. pratense*	Kirichenko N.	SIF	5.VII.2015	Russia	Krasnoyarsk, Yenisei, Skala Berkut,	55.97	92.65	173
	Parectopa robiniella									
84	A (1), CNCLEP00132251, [MIC 6972 ♀]	*Robinia sp.*	McDunnough J.	CNC	14.IV.1946	Canada	Nova Scotia, Smiths Cove	44.61	-67.70	31
85	A (1), CNCLEP00121057, [MIC 6907 ♀]	*Robinia sp.*	McDunnough J.	CNC	15.IV.1946	Canada	Nova Scotia, Smiths Cove	44.61	-67.70	31

* barcoding attempted but failed.

— no data.

[1]**Collections:**

ANSP – Academy of Natural Sciences of Philadelphia, Philadelphia, Pennsylvania, U.S.A.

CNC – Canadian National Collection of Insects, Arachnids, and Nematodes, Agriculture and Agri-Food Canada, Ottawa, Ontario, Canada.

SIF – Sukachev Institute of Forest, Siberian Branch of the Russian Academy of Sciences, Krasnoyarsk, Russia.

MSNV – Museo Civico di Storia Naturale, Verona, Italy.

USNM – National Museum of Natural History, Smithsonian Institution, Washington, D.C., U.S.A.

WSDA – Washington State Department of Agriculture, Olympia, Washington, U.S.A.

Table S3. Diagnostic substitutions in COI barcode sequences of *Micrurapteryx caraganella* and *M. gradatella*.

**Position**	**10**	**13**	**14**	**22**	**25**	**31**	**49**	**79**	**82**	**133**	**139**	**154**	**160**	**169**
M. caraganella	C	T	T	G	T	G	A	A	C	T	T	T	A	T
M. gradatella	T	C	C	A	C	A	T	G	T	C	C	C	G	A

**Position**	**211**	**212**	**220**	**241**	**266**	**271**	**277**	**281**	**283**	**325**	**334**	**337**	**346**	**355**
M. caraganella	G	T	T	A	C	C	C	C	A	C	A	A	T	T
M. gradatella	A	C	C	G	T	T	A	T	G	T	G	G	C	A

**Position**	**358**	**367**	**382**	**385**	**386**	**400**	**407**	**409**	**421**	**427**	**442**	**445**	**475**	**487**
M. caraganella	C	C	C	T	C	C	T	A	T	C	T	C	C	A
M. gradatella	T	T	A	C	T	T	C	T	A	T	C	T	T	T

**Position**	**499**	**500**	**517**	**536**	**538**	**541**	**542**	**544**	**557**	**550**	**553**	**557**	**589**	**640**
M. caraganella	T	C	A	C	T	A	T	A	C	A	T	C	C	C
M. gradatella	A	T	G	T	A	T	C	T	A	G	A	T	T	T

Table S4. Diagnostic substitutions in histone H3 and 28S sequences of *Micrurapteryx caraganella* and *M. gradatella*.

	**Histone H3**		
**Position**	**43**	**55**	**259**
M. caraganella	G	G	A
M. gradatella	A	A	G

	**28S**	
**Position**	**109**	**536**
M. caraganella	A	G
M. gradatella	G	A

Table S5. Genital characters of *Micrurapteryx* and *Parectopa* extracted from Vári's (1961) generic descriptions.

Character	*Micrurapteryx*	*Parectopa*
**Male genitalia**		
tegumen	weakly sclerotized	moderate, membranous
tuba analis	long, membranous	moderate, membranous
valvae	moderate, longitudinally cleft	rather large, parallel-sided, truncate
costa	straight	—
cucullus	projecting	long-haired, harpe and ventral margin for a short distance set with fine, irregular, sclerotized teeth
valvula	with well sclerotized teeth	—
vinculum	strongly dilated dorsally	narrow
saccus	short pointed	very short
aedeagus	rather long, slender, with one cornutus	moderate, slender, a little thickened beyond middle, curved, apex long and narrow, no cornuti
7th sternite	—	with slender prong laterally
coremata	one pair, long, slender	—
**Female genitalia**		
papillae anales	moderate, oblong	rather small, oblong, weakly haired
posterior apophyses	moderate, straight	short, straight
anterior apophyses	wide at base, long-pointed, straight	2x longer than p.a., very slender
sterigma	laterally projecting	simple
ostium	wide, oblong	moderate, almost circular
antrum	tubular, slightly tapering, sclerotized	short, weakly sclerotized, ductus seminalis from just beyond antrum
ductus bursae	moderate, membranous	moderate, weakly sclerotized, membranous towards corpus bursae
corpus bursae	moderate, membranous, with two small scobinated patches in centre	moderate, membranous, no signa
