# Supplementary figures and images for: Systematics and biology of some species of Micrurapteryx Spuler (Lepidoptera, Gracillariidae) from the Holarctic Region, with re-description of M. caraganella (Hering) from Siberia
Source: Zookeys. 2016 Apr 11;(579):99–156. doi: 10.3897/zookeys.579.7166 (PMC4829971; doi:10.3897/zookeys.579.7166)

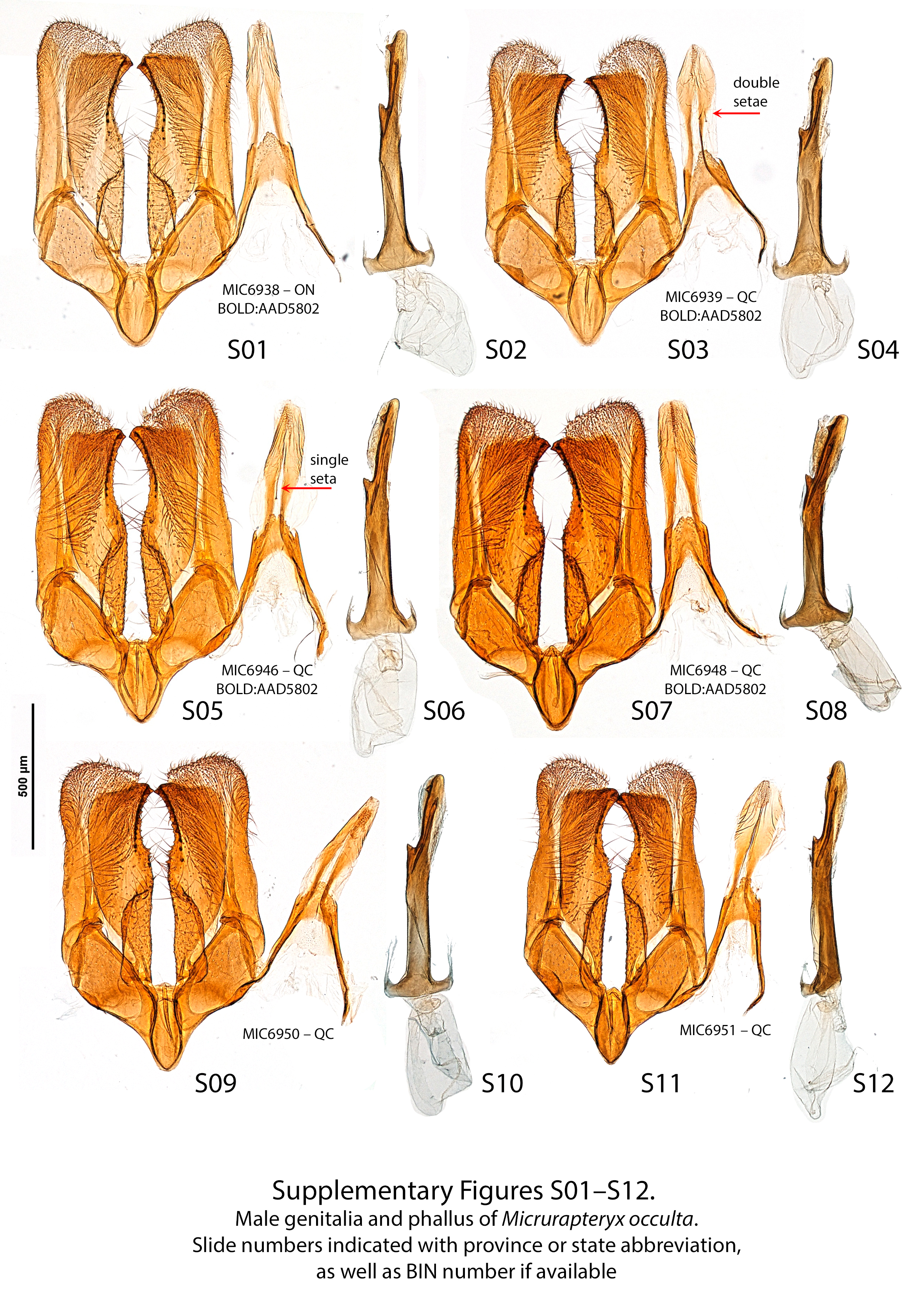

Supplement: Supplementary material 2 — Figs S01–S12 [file zookeys-579-099-s002.jpg]

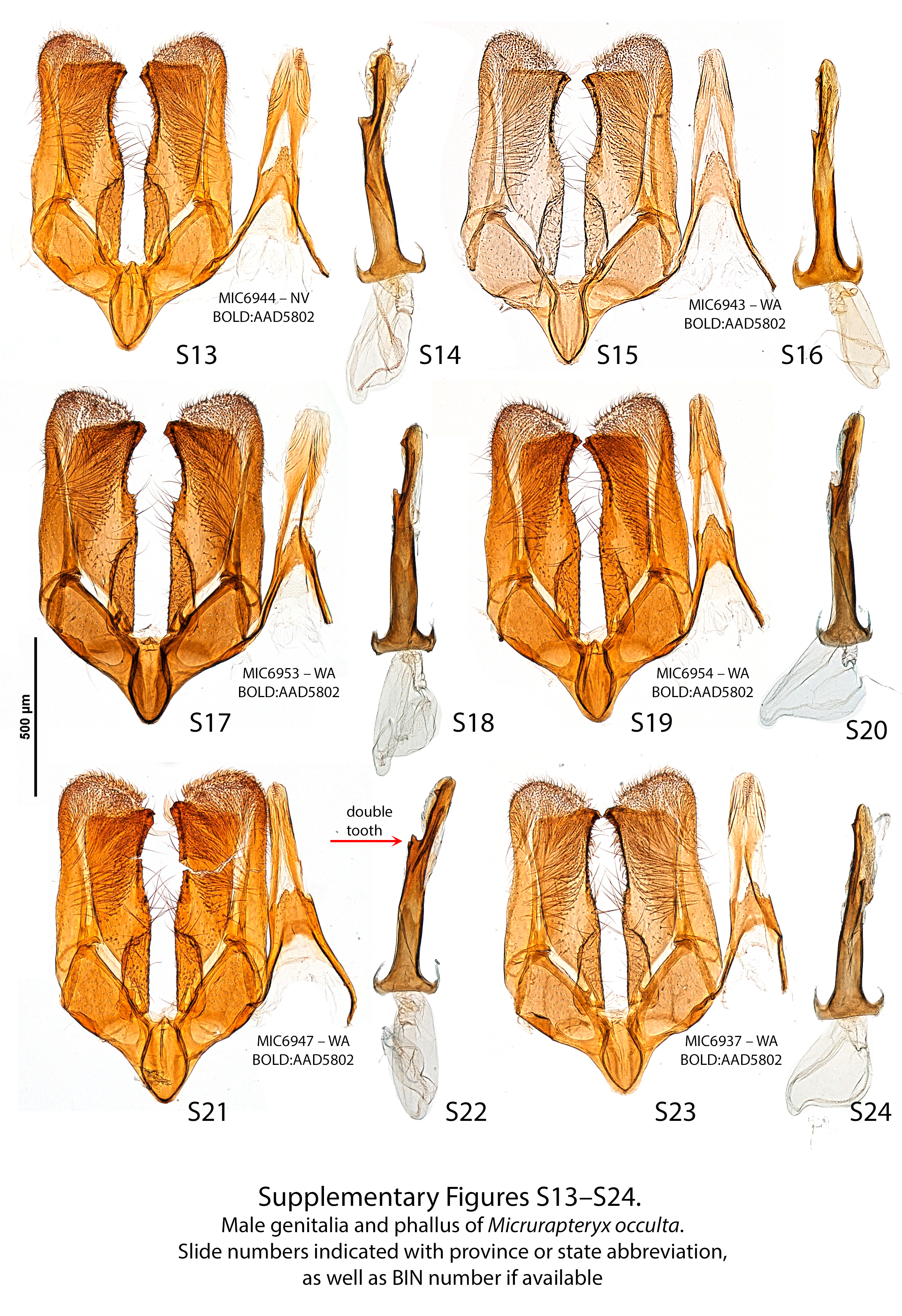

Supplement: Supplementary material 3 — Figs S13–S24 [file zookeys-579-099-s003.jpg]

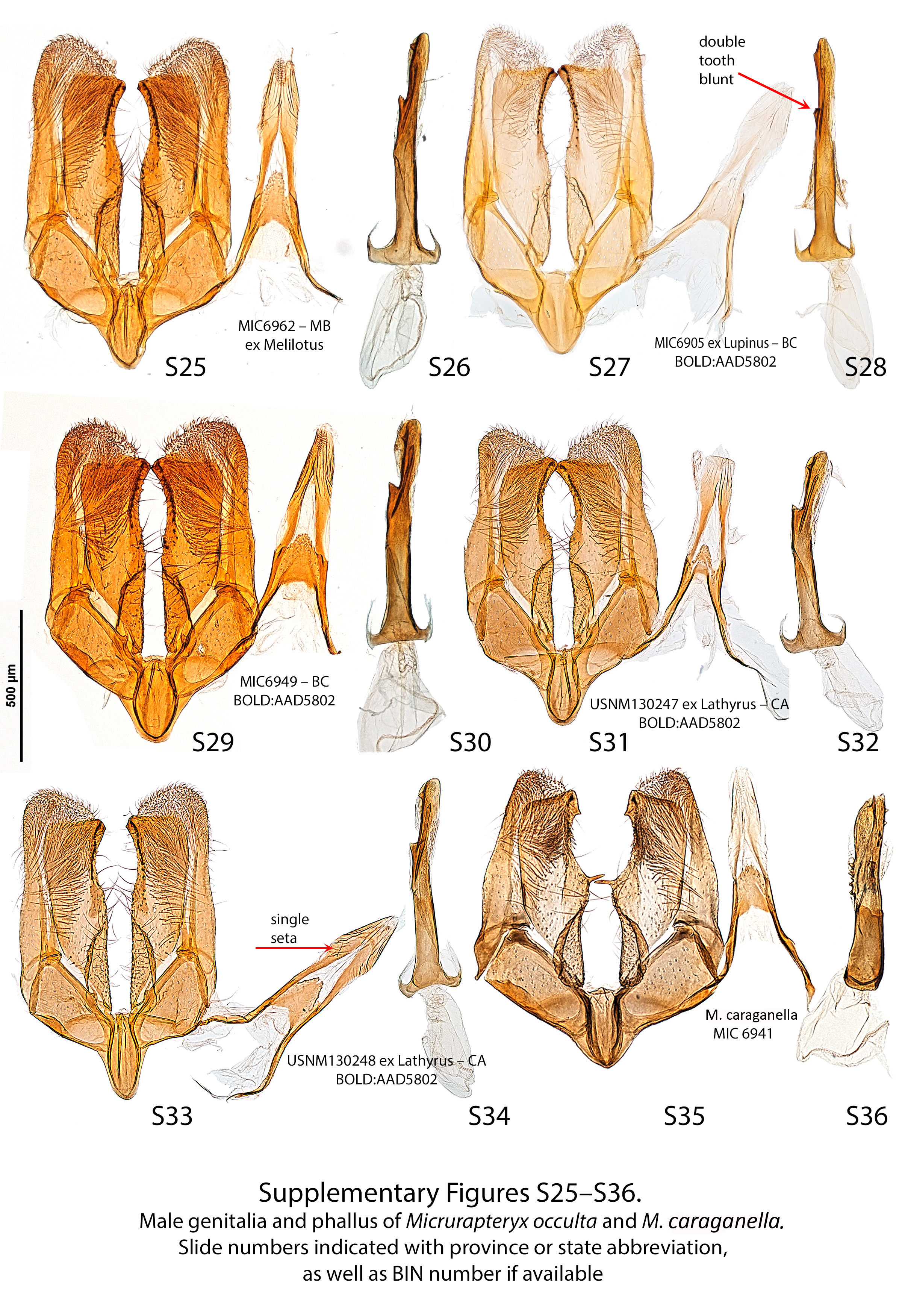

Supplement: Supplementary material 4 — Figs S25–S36 [file zookeys-579-099-s004.jpg]

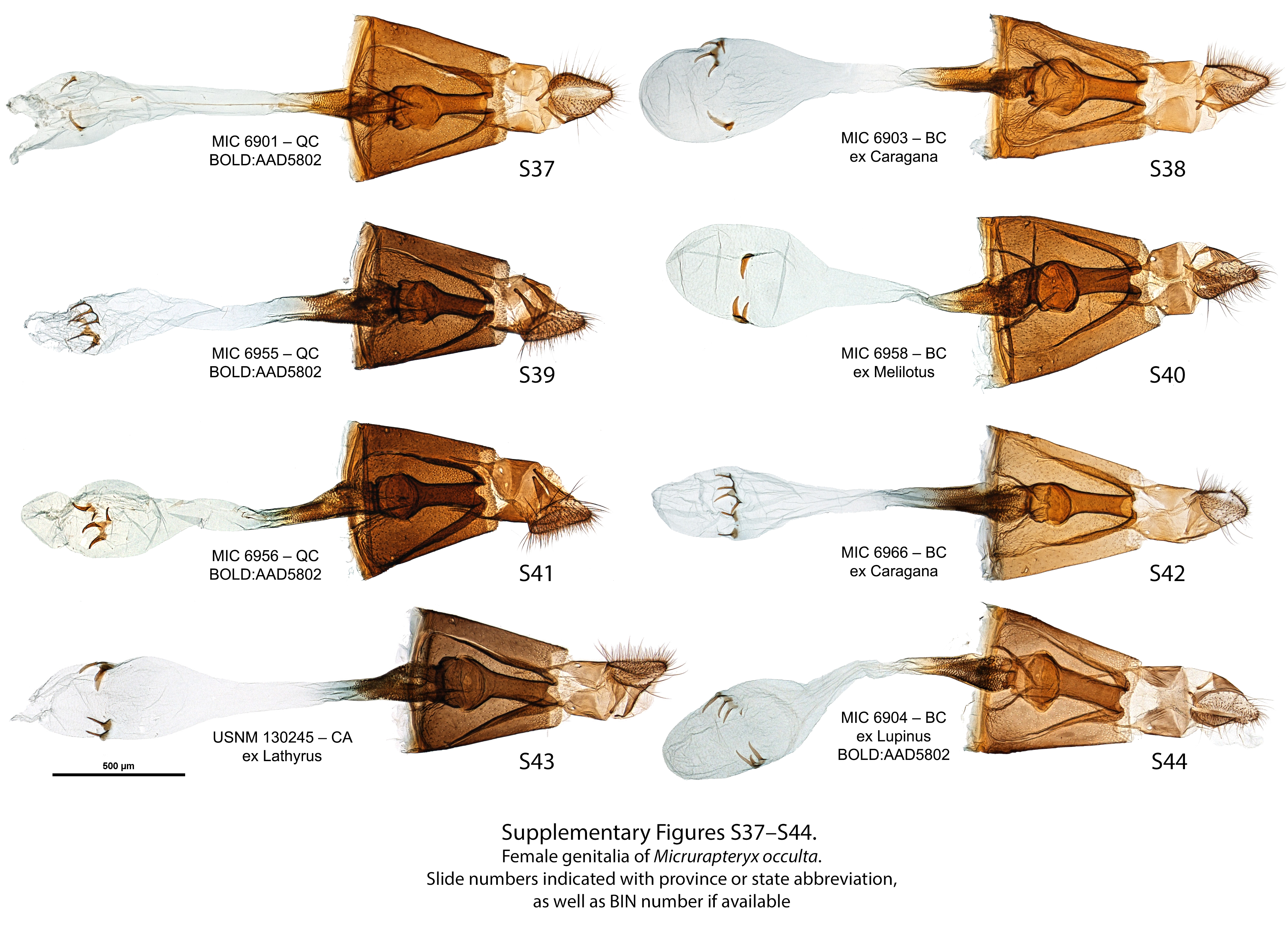

Supplement: Supplementary material 5 — Figs S37–S44 [file zookeys-579-099-s005.jpg]

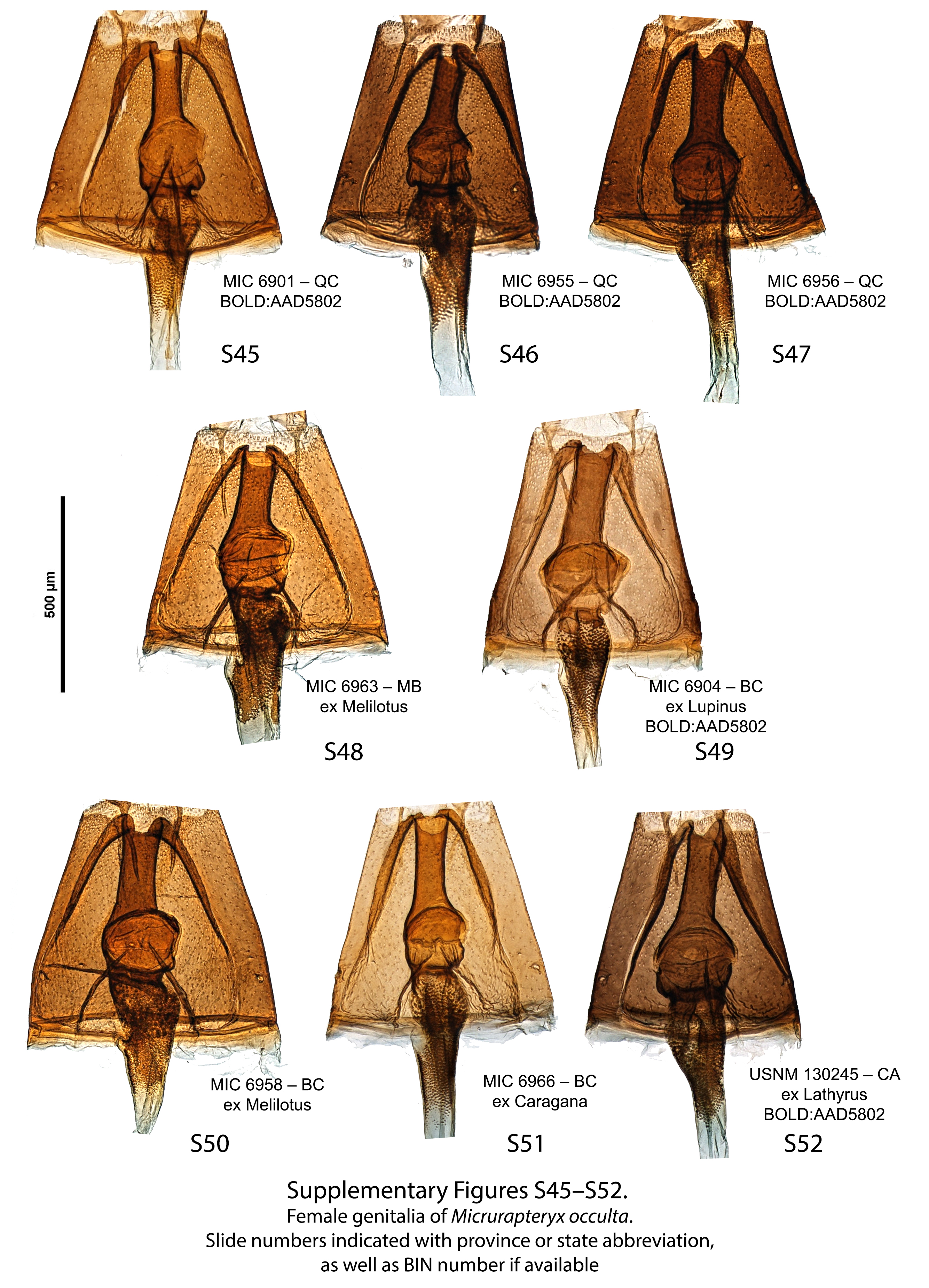

Supplement: Supplementary material 6 — Figs S45–S52 [file zookeys-579-099-s006.jpg]
